# Electoral fortunes reverse, mindsets do not

**DOI:** 10.1371/journal.pone.0208653

**Published:** 2018-12-14

**Authors:** Theodore Samore, Daniel M. T. Fessler, Colin Holbrook, Adam Maxwell Sparks

**Affiliations:** 1 Department of Anthropology and Center for Behavior, Evolution and Culture, University of California Los Angeles, Los Angeles, California, United States of America; 2 Department of Cognitive and Information Sciences, University of California Merced, Merced, California, United States of America; Saint Peter's University, UNITED STATES

## Abstract

Conservatives and liberals have previously been shown to differ in the propensity to view socially-transmitted information about hazards as more plausible than that concerning benefits. Given differences between conservatives and liberals in threat sensitivity and dangerous-world beliefs, correlations between political orientation and negatively-biased credulity may thus reflect endogenous mindsets. Alternatively, such results may owe to the political hierarchy at the time of previous research, as the tendency to see dark forces at work is thought to be greater among those who are out of political power. Adjudicating between these accounts can inform how societies respond to the challenge of alarmist disinformation campaigns. We exploit the consequences of the 2016 U.S. elections to test these competing explanations of differences in negatively-biased credulity and conspiracism as a function of political orientation. Two studies of Americans reveal continued positive associations between conservatism, negatively-biased credulity, and conspiracism despite changes to the power structure in conservatives’ favor.

## Introduction

Most American parents do not allow their children to collect unwrapped Halloween candy, believing falsely that malicious agents sometimes give out poisoned treats [1]. A few Americans think that the tragic 2012 mass murder at Sandy Hook Elementary School was a hoax [2]. What underlies variation in the propensity to believe various types of information, a dimension of individual difference that powerfully ramifies through the personal, social, and political domains of contemporary life? One account holds that such variation stems from intrinsic psychological differences in the assessment of threats versus opportunities; another account instead suggests the pattern is a reaction to impermanent features of the political power structure. Here, we take advantage of the consequences of the 2016 U.S. elections to test these two hypotheses.

When the world is dangerous, it generally pays to be highly cognizant of hazards, and to stick to tried and true behavioral patterns. However, in the absence of danger, such an approach wastes resources on unnecessary defenses while foregoing potentially beneficial opportunities. Conversely, when the world is safe, it often pays to reduce vigilance and to experiment with new behaviors; however, when dangers emerge, this approach exacerbates vulnerability. Because people vary in their ability to confront and bear the costs of threats, the relative dangerousness of any given situation is a function of both that environment and the individual [3]. Accordingly, neither strategy is invariably optimal; rather, their relative utility depends on the intersection of the local environment and individual capabilities, rendering both strategies contextually viable. Because environments and individuals vary substantially along these dimensions, natural selection can be expected to have maintained variation in perceptions of the dangerousness of the world and reactivity to indications of threat.

The contemporary political constructs of ‘‘conservative” and ‘‘liberal” echo an abiding division in social preferences related, respectively, to the maintenance of traditional order, and to the pursuit of change [4]. These terms can be understood as anchoring a spectrum summarizing multidimensional variation in political cognition and behavior. In actual political contests, historical and cultural vicissitudes result in coalitions with shifting alliances and factions. Nevertheless, despite path-dependent complexities of coalition formation and the noisiness of psychological measures, gradients are nonetheless evident along the conservative-liberal dimension, revealing divergent information-processing styles that lead like-minded conservatives or liberals to identify with, and assort into, their respective coalitions [5]. Critically, conservatism has been theorized to in part reflect a worldview that values traditionalism and the cultural in-group as safeguards against a world perceived to be more dangerous; conversely, liberalism can be understood as reflecting a worldview in which, against a backdrop of the perception of the world as relatively safe, change and cultural plurality constitute opportunities [6–13]. Hence, conservativism predicts elevating, and liberalism predicts depressing, the perceived significance of risks relative to opportunities [13], [14]. Consonant with the above, researchers have found that conservatism is associated with greater negativity and threat bias [13], [15–19], leading threat-related information to be more salient for conservatives compared to liberals. Right-wing authoritarianism, a personality construct which indexes attitudes toward social change [20], [21], is also associated with beliefs in a dangerous world [21], and likely plays an important role in the relationship between threat bias and conservatism [13].

The tendency to readily perceive and be highly reactive to possible hazards can be regarded as a manifestation of negativity bias, a pattern of decision-making exhibited by diverse organisms [22–25]. Considering its widespread phylogenetic distribution, negativity bias likely serves an adaptive purpose. If, as appears to often be the case, a given hazard is more imminent than is an opportunity to obtain a benefit of comparable fitness magnitude, and if, as is also often true, the former precludes the latter, then failing to act on a potential benefit will generally have lower fitness costs than failing to act on a potential threat. Correspondingly, selection favors the evolution of mechanisms that tend to invest more in attending to perceived hazards versus other kinds of information [24], [25]. In humans, negative information is more salient, memorable, and emotionally evocative than positive information [24–26], presumably exerting a stronger influence on behavior, and, ultimately, fitness.

While negativity bias applies when organisms assess any information, human reliance on culture raises special considerations. We should expect humans to possess specific adaptations tailored to the acquisition of culture [27], including psychological mechanisms related to assessing the value and reliability of socially transmitted information. The causal efficacy, and sometimes even the utility, of cultural information is frequently opaque for the learner, and, in some cases, for experts as well [28], [29]. As a consequence, individuals can often benefit by being credulous of socially transmitted information, profiting from culture knowledge despite not understanding why or even if it is effective [14], [30]. However, excessive credulity is detrimental, as overly credulous individuals risk exploitation and other costs of acting on false information [31]. Consequently, individuals must calibrate credulity to the costs and benefits of belief versus non-belief. While the source of information is a key factor in epistemic vigilance [32], holding this consideration constant, not all types of information are created equal. When socially transmitted information concerns hazards, mistaken incredulity is often costlier than mistaken credulity because of the asymmetry between the costs of unnecessary vigilance and the much larger costs of injury or death after disregarding an actual threat [14], [30]. In contrast, when socially transmitted information concerns benefits, there is no obvious systematic asymmetry in costs between erroneous incredulity and erroneous credulity. Hence, ceteris paribus, people should more readily believe socially transmitted information when that information addresses hazards rather than benefits, a prediction largely borne out in prior studies [14], [30] (see also [33–35]).

Negatively-biased credulity for socially transmitted information can be understood as a manifestation of the logic of general negativity bias. Further, because threats frequently co-occur, prior evidence (or prior belief) that the world is a more dangerous place also predicts higher negatively-biased credulity [30]. Accordingly, given that conservatism is correlated with both belief that the world is relatively dangerous and greater orientation towards the possibility of further threats, negatively-biased credulity should correlate with conservatism. Indeed, Fessler and colleagues [14] found that, in the U.S., conservatism is associated with a greater propensity to believe socially transmitted information about hazards relative to benefits. Per the functional logic presented above, the authors attributed this to endogenous and stable differences in threat reactivity and dangerous-world beliefs that underlie individuals’ tendencies toward conservatism or liberalism. Critically, however, this work was conducted at a particular point in U.S. political history.

While not explicitly addressing threat bias, research on the tendency to endorse conspiracy theories—which can be thought of as a special category of socially transmitted hazard information—offers a divergent explanation to the above. Contrary to the threat-reactivity model of partisan differences, researchers have argued that the propensity to believe conspiracy theories does not inherently correspond with political orientation [36], [37]. Instead, any observed differences in conspiracism between conservatives and liberals [38–40] are thought to be a response to the distribution of power in the political hierarchy at the given time [38], [41], [42].

By this account, political parties (or branches thereof) that are out of power—termed political “losers” [42]—directly or indirectly promote threat-related conspiracy theories in order to increase group cohesion and direct ire and vigilance at opposing political actors, or “winners” [38], [41], [42]. By extension, any differences between conservatives and liberals in negatively-biased credulity would be attributable not to intrinsic differences between the two, but rather to the fact that, at any given time, they occupy different rungs in the political hierarchy, thus generating impermanent variation in threat bias that mirrors changes in the power structure. Indeed, following publication of Fessler and colleagues’ 2017 paper on the association between conservatism and negatively-biased credulity [14], Joseph Uscinski, one of the principal proponents of the power-hierarchy account, noted that “Until [Fessler et al.’s] findings have been replicated under the changed circumstances of a Republican victory, they should be greeted with caution.” [43].

The above two contrasting explanations for observed differences between conservatives and liberals generate divergent predictions regarding the stability of patterns of hazard credulity in general, and conspiracism in particular. If these differences reflect endogenous features adapted to calibrate an individual’s trade-off between unnecessary precaution and obliviousness to actual hazards, they ought to be consistent across time and robust to changes in the power structure. Conversely, if threat bias is a strategic response to a lack of political power, negatively-biased credulity and political orientation ought to track changes in the power structure.

The 2016 U.S. elections provided an opportunity for testing between the two competing accounts outlined above. The party generally associated with a liberal political orientation (the Democratic Party) held considerable power when much prior research on threat bias and political orientation was conducted, hence the two competing hypotheses generated similar predictions during this period. However, the Democrats suffered severe defeats across all three branches of the federal government in 2016; the party associated with conservatism (the Republican Party) was then firmly in control for the first time since 2006. If the relationship between political orientation and informational attitudes were to reverse in the wake of the 2016 elections—that is, if liberalism were to become positively correlated with negatively-biased credulity—then this would support the contention that differential processing of threat-related information is driven by the prevailing power structure. In contrast, if conservatism were to continue to predict negatively-biased credulity even after the 2016 election, this would support the conclusion that political orientation reflects stable individual differences regarding the salience and believability of threat-related information. Lastly, occupying the middle ground between these two extremes, if the relationship between political orientation and negatively-biased credulity were to weaken, but not reverse, following the 2016 election, this would suggest that both hypotheses are correct. Relatedly, the 2016 elections and events thereafter provide an opportunity to test the specific thesis from which the aforementioned social structural account of differences in negatively-biased credulity is derived, namely the notion that conspiracy beliefs in general flourish among supporters of the party currently out of power in part because these individuals find such ideas plausible. If it is true that those who are currently at the losing end of contests over political power display greater conspiracism, then, in contrast to the currency of conspiracy beliefs among conservatives prior to the election, those who affiliate with the Democratic Party should now be the ones inclined to see shadowy forces at work.

Taking advantage of the changes entailed by the 2016 election, we sought to methodologically replicate—and extend—the research that Fessler et al. [14] previously carried out prior to said election. By anchoring our project to Fessler et al.’s studies, we were able to create a between-subjects before-and-after design, while also adding new measures to secondarily address the competing hypothesis. We thus conducted two online studies of Americans in 2017 in order to i) test between the stable individual differences explanation of the relationship between political orientation and negatively-biased credulity and the social-structural account, and ii) evaluate the thesis that conspiracism is driven by losing out in contests over political power. Study 1 was conducted six months after the 2016 election. Because shifts in participants’ impressions of the power structure may lag behind actual changes to the political hierarchy at the federal level, Study 2 was conducted 13 months following the 2016 election, immediately after the senatorial special election in Alabama, an event receiving extensive media coverage that underscored the power dynamic. Further, although most participants were presumably aware of the results of the 2016 election, there are likely to be individual differences in how salient both said event and the outcome of the Alabama special election were in updating participants’ priors about their preferred party’s place in the political hierarchy. Therefore, in Study 2, we also collected information about participants’ confidence in their preferred party’s political power and future success.

## Materials and methods

All study protocols reported in this paper were approved by the University of California, Los Angeles Office of the Human Research Protection Program. Informed consent was obtained before participation. Because the data was analyzed anonymously, the Office of the Human Research Protection Program granted a waiver of signed consent. Complete surveys, datasets, and analysis code are available for both studies, as is a preregistration of predictions and methods for Study 2, at https://osf.io/v8n6g/?view_only=aab4526f905247f0aa648ccd92ccc13a.

### Study 1

Based on results in Fessler et al. [14], a final sample size of 450 was targeted. To account for attrition and exclusion, 517 U.S. participants were recruited in April of 2017 via the Prolific recruitment platform in exchange for $1.25. Using Prolific’s system, the sample was evenly divided between self-identified Republicans and self-identified Democrats. Data were prescreened for repeat participation, English fluency, minimal completeness, and answering “catch questions”. The final sample consisted of 449 adults (48% female; 80% white) ranging in age from 18 to 78 (*M* = 32.2, *SD* = 11.8). In selecting our target sample size, we simply emulated Fessler et al. [14], and did not conduct a power analysis of this study. Nevertheless, a post hoc analysis based on the effects from Fessler et al.’s Study 1 suggests that the present study would have power = .34 to detect the predicted relationship between political orientation and negatively-biased credulity at *p* = .05; likewise, a post-hoc power analysis informed by the effect size observed in Fessler et al.’s Study 2 indicates that the present study would have power = .74.

#### Credulity ratings

Participants first completed Fessler et al.’s [14] credulity scale, comprised of 16 statements partitioned into eight domains. Each domain includes both a hazard statement and a benefit statement (e.g., “*Eating carrots results in significantly improved vision*”, “*Kale contains thallium*, *a toxic heavy metal*, *that the plant absorbs from soil*”); these statements were designed to be essentially apolitical. Participants rated both the truthfulness of the statements along a 7-point Likert scale (1 = *I’m absolutely certain this statement is FALSE*; 7 = *I’m absolutely certain this statement is TRUE*) and the perceived magnitude of the hazard or benefit along a 7-point Likert scale (1 = *The benefit [hazard] described in this statement is SMALL*; 7 = *The benefit [hazard] described in this statement is LARGE*). For each of the eight domains within the scale, the magnitudes of the hazard and benefit statements within a domain were designed to be approximately equal. Statements were presented in random order. Credulity bias was operationalized as the difference between the mean believability ratings for hazard-related and benefit-related items.

#### Conspiracism

Next, participants answered the five-item (α = .80) Conspiracy Mentality Questionnaire (CMQ) [39]. Participants rated the truthfulness of five statements along 1–11 scales (1 = *Certainly not true*; 11 = *Certainly true*). By design, these items do not probe whether participants subscribe to any particular conspiracy theories, but rather assess their tendency toward conspiracy thinking in general (e.g., *“I think that events which superficially seem to lack a connection are often the result of secret activities”*). This has the advantage both that it minimizes the influence of any specific prior beliefs held by a participant, and it sidesteps issues as to the inherent plausibility of a particular belief (an important feature given that some conspiracy theories in recent circulation in the U.S. are prima facie highly implausible).

#### Political orientation

Finally, following methods employed by Fessler et al. [14], political orientation was measured using a slightly updated form of Dodd et al.’s [44] version of Wilson and Patterson’s [45] issues index; participants indicated agreement, disagreement, or uncertainty regarding 29 contemporary political issues, amongst which 14 are generally favored by conservatives (e.g., “death penalty”, “small government”), and 15 generally favored by liberals (e.g., “abortion rights”, “welfare spending”) (α = .90). For each topic, agreement was scored as +1, disagreement as -1, and uncertainty as 0. Liberal topics were reverse coded, hence increasing positive values indicated greater conservatism, and increasing negative values indicated greater liberalism. Topics in this index variably addressed issues relating to social, militaristic, and economic political values. Participants were also asked to identify their political affiliation by party. Demographic items followed.

### Study 2

On December 15^th^, 2017, three days after the Alabama special senatorial election, 498 U.S. participants were recruited via MechanicalTurk.com in exchange for $0.80. Using Mechanical Turk’s qualification system, the sample was evenly balanced between self-identified Republicans and self-identified Democrats. Data were pre-screened for repeat participation, English fluency, minimal completeness, and answering “catch questions”. The final sample consisted of 436 adults (60% female; 83% white) ranging in age from 19 to 74 (*M* = 41.7, *SD* = 12.8). As in Study 1, in selecting our target sample size for Study 2 we emulated Fessler et al. [14], and did not conduct power analyses. Nevertheless, a post-hoc analysis informed by the effect size observed in our Study 1 indicates that our sample for Study 2 had power = .96 to detect the predicted relationship between political orientation and negatively-biased credulity.

#### Credulity ratings

Participants first completed the 16-item credulity scale as in Study 1.

#### Conspiracism

Participants then answered the 5-item CMQ (α = .86) as in Study 1.

#### Political confidence

Next, to probe individual differences in subjective impressions of one’s place in the political power hierarchy, participants rated how confident they were in their preferred political party’s future success (e.g., *“I think that the political party I prefer will control Congress after the 2018 midterm elections”*). Participants rated four such political confidence items (α = .82) on 1–11 scales (1 = *Certainly not true*; 11 = *Certainly true*). Relatedly, to get a better sense of how changes to the power structure influence the relationship between negatively-biased credulity and political orientation, information was collected on participants’ preferred political party, preferred candidate in the 2016 general presidential election, and the degree to which participants identified with an array of major political parties.

#### Political orientation

Political orientation was then assessed using the same modified Dodd et al. [44] version of the Wilson and Patterson [45] issues index employed in Study 1, which continued to demonstrate internal consistency (α = .91).

#### Special election items

To better measure the effects of changes to the political power structure on the variables of interest, we also asked participants questions about the 2017 senatorial special election in Alabama, which took place immediately prior to Study 2. Participants were asked to identify who won the special election; whether their preferred candidate won; how aware they were of the special election; how concerned they were about the outcome of the special election; and how much media they consumed about the special election. Finally, demographic data were collected.

## Results

### Negatively biased credulity

Following Fessler et al [14], we weighted participants’ credulity ratings using their assessments of the perceived magnitude of those statements. Consistent with both the theory of negatively-biased credulity and previous findings [14], [30], in Study 1, participants’ weighted credulity scores were higher for hazards than for benefits (hazard: *M* = 12.15, benefit: *M* = 11.20), *t*(881.98) = 2.89, *p* < .001; these two types of credulity were correlated, *r* = .53, *p* = < .001. The same was true in Study 2 (hazard: *M* = 13.08, benefit: *M* = 11.63), *t*(877.60) = 4.25, *p* < .001; the two types of credulity were again correlated, *r* = .42, *p* = < .001.

### Political orientation and credulity

**Fig 1:**
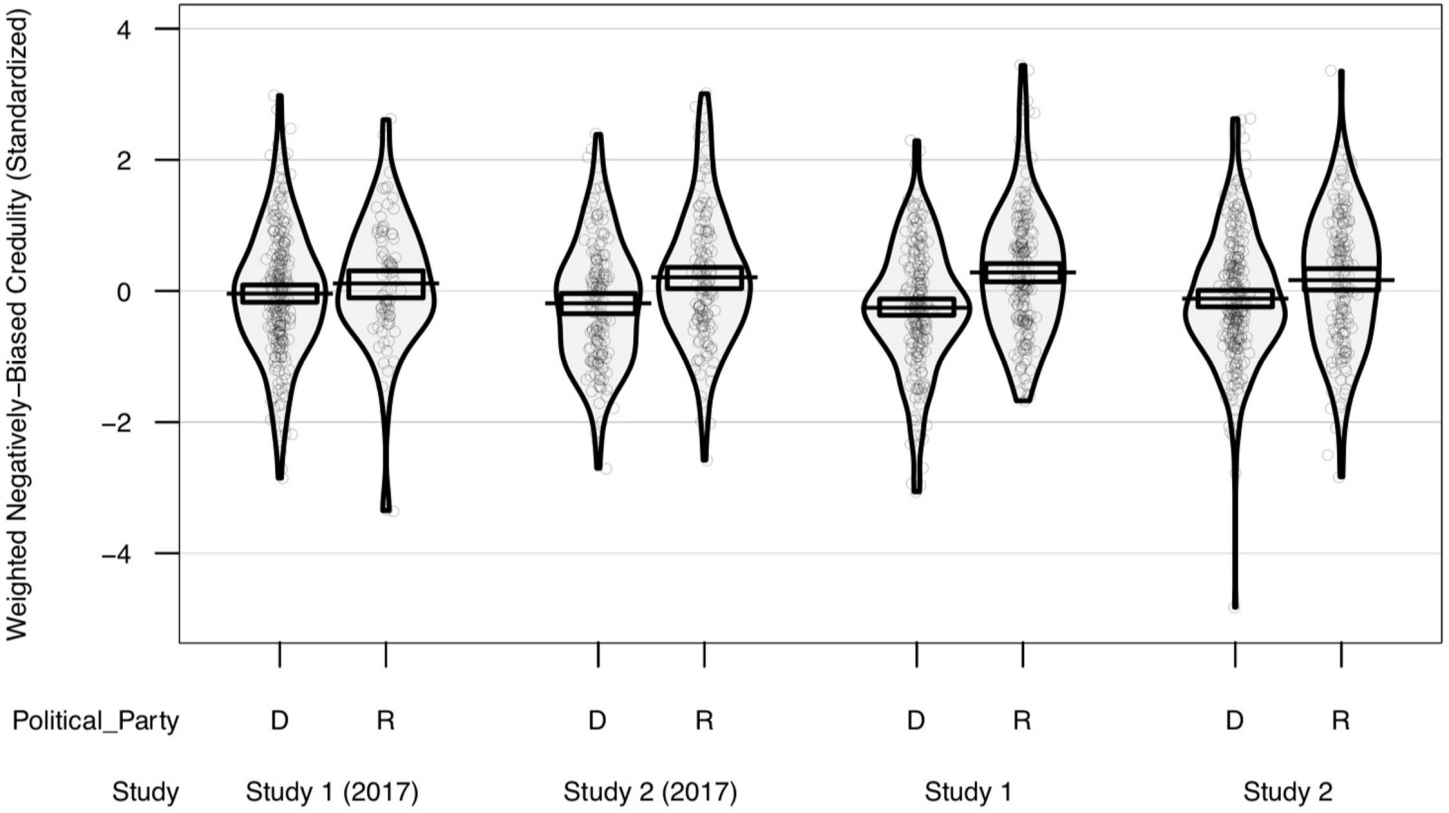
Negatively-biased credulity and political party. Negatively-biased credulity as a function of political party affiliation, comparing current results from Fessler et al.’s 2017 paper [14] with those from the current studies. Scatterplot points are individual scores, jittered along the (meaningless) horizontal axis to reduce overlap. Only data from self-identified Democrats (D) and Republicans (R) included. Beans show smoothed density of data points. Bars and boxes represent means and Bayesian 95% highest density intervals, respectively.

To adjudicate between the hypotheses, for both Studies 1 and 2, we fit linear models with the difference between weighted hazard and benefit credulity as the response, and conservatism and demographics as predictors. Even after substantial changes in the federal political power hierarchy, conservatives exhibited greater credulity for statements concerning hazards relative to those concerning benefits, with effects at least as large as Fessler et al [14] (Table 1; see S1 Supporting information for full models), an effect robust to the removal of any one credulity item, across a suite of models with alternative predictors sets (e.g., political orientation subscales), or using unweighted credulity (see S1 Supporting information). Further, in both Studies 1 and 2, using party affiliation instead of political orientation produces similarly interpretable results of similar, if slightly larger, size to Fessler et al [14] (see Fig 1, and S1 Supporting information). Models specific to hazard or benefit credulity are presented and discussed in S1 Supporting information; conservatism robustly predicts weighted hazard credulity.

**Table 1:** Negatively-biased credulity and political orientation. Results of models using summary measure of political orientation and demographic variables to predict difference between weighted hazard credulity and weighted benefit credulity.

study	term	b	95% CIs for b	Beta	p
study1	(Intercept)	2.00	[-0.29, 4.29]	0.00	0.09
	conservatism	2.62	[1.54, 3.69]	0.23	0.00
study2	(Intercept)	3.82	[1.4, 6.24]	0.00	0.00
	conservatism	2.75	[1.58, 3.93]	0.22	0.00
	sex = male	-1.35	[-2.39, -0.32]	-0.12	0.01

*Note*. Model fit statistics for Study 1: adjusted R^2 = .04, F(6, 421) = 4.15. Model fit statistics for Study 2: adjusted R^2 = .04, F(6, 421) = 4.15. Predictors with p > .10 not displayed.

### Conspiracism

**Fig 2:**
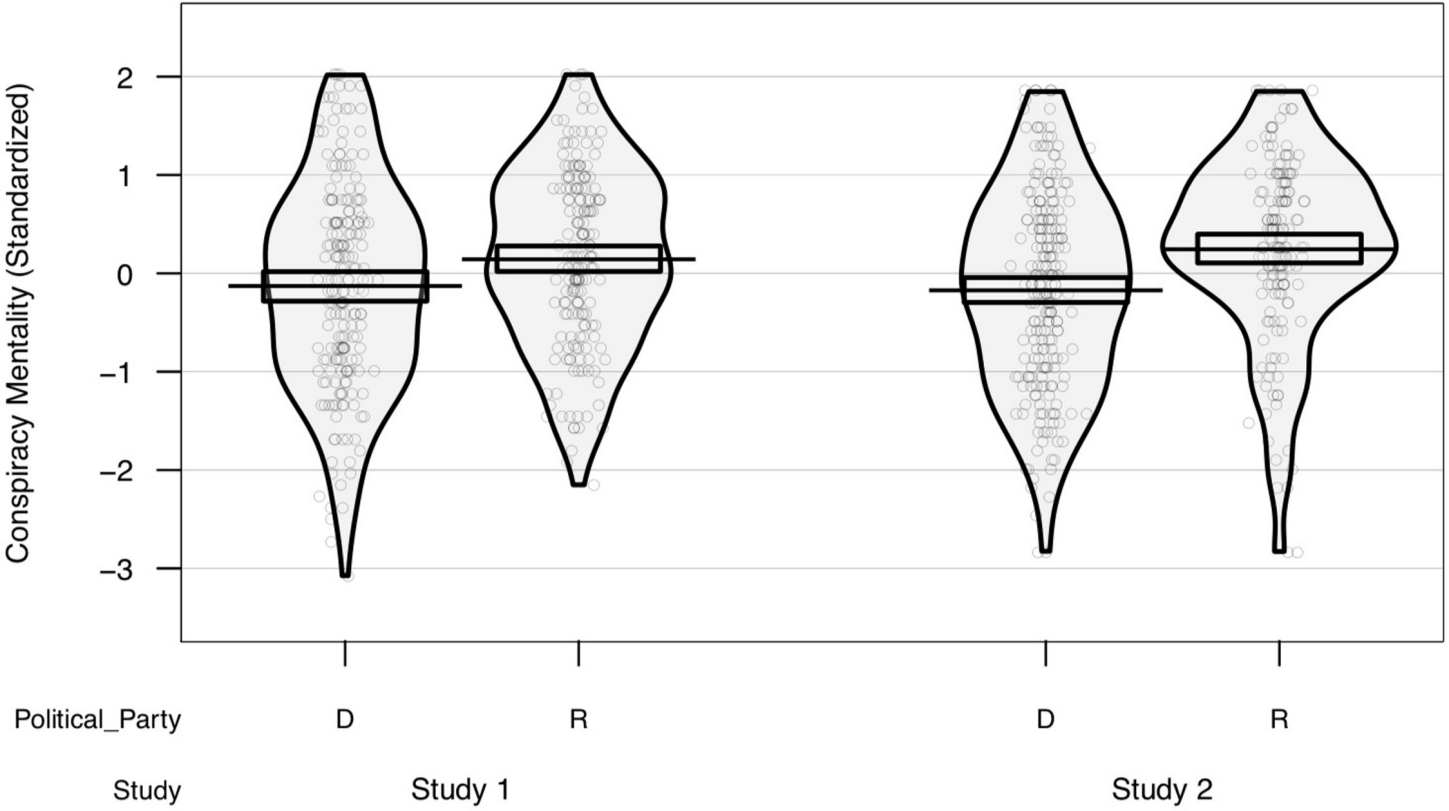
Conspiracism and political party. Conspiracy mentality as a function of political party affiliation. Scatterplot points are individual scores, jittered along the (meaningless) horizontal axis to reduce overlap. Only data from self-identified Democrats (D) and Republicans (R) included. Beans show smoothed density of data points. Bars and boxes represent means and Bayesian 95% highest density intervals, respectively.

Conservatism positively predicted conspiracy mentality in both Studies 1 and 2, *r*s .21-.31, *p*s < .001. Similarly, party affiliation produced analogous results (see Fig 2). Further, hazard credulity was positively associated with conspiracism (both studies *r* = .36, *p* < .001), suggesting possible parallel effects of negativity bias on both phenomena. When included in the same model, both negatively-biased credulity (β s .13-.14, *p*s < .05), and conservatism (βs .19-.33, *p*s < .01) were significant predictors of conspiracism (see S1 Supporting information for full models). Omitting the one item from the CMQ that references “government” does not change the results pattern (see S1 Supporting information).

### Political confidence

**Fig 3:**
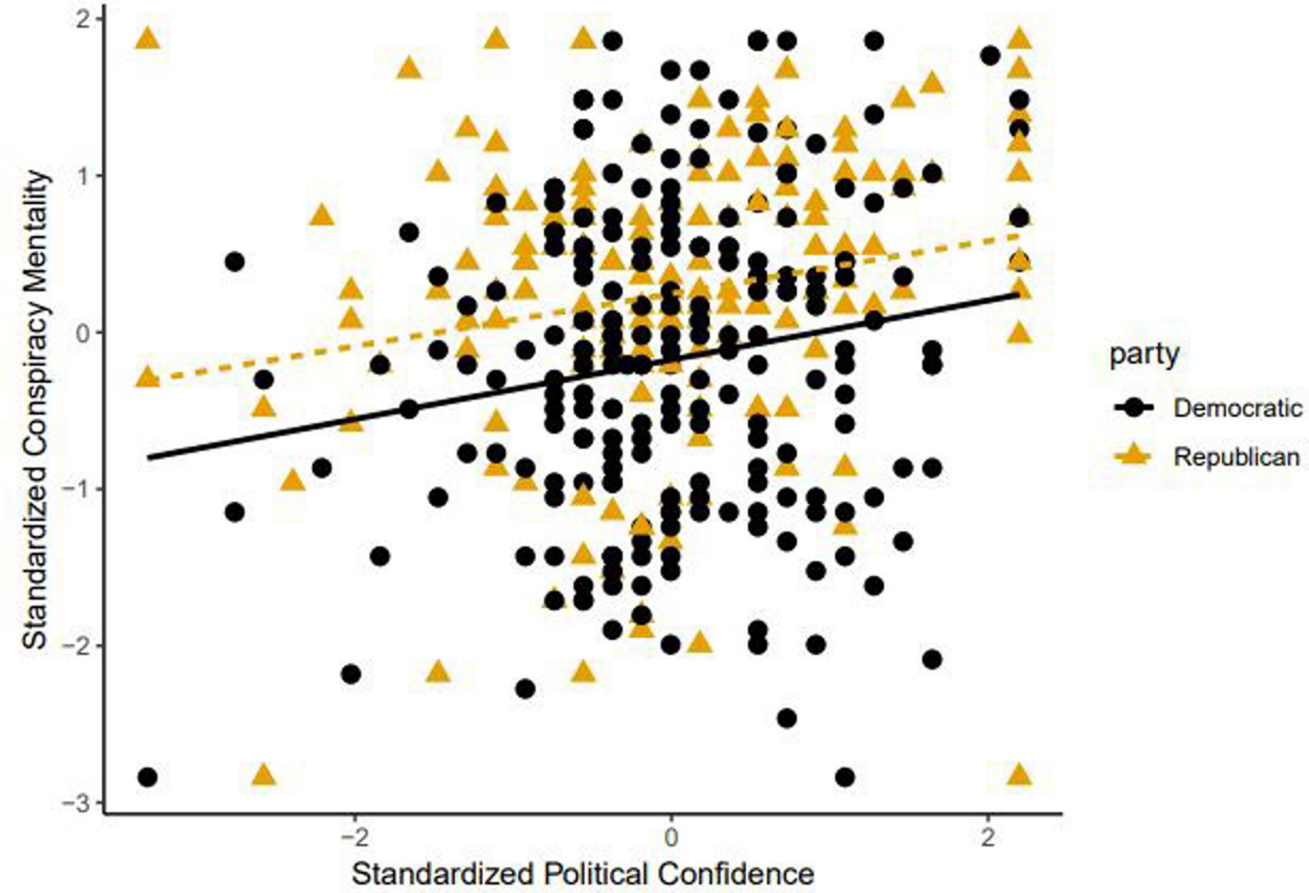
Conspiracism and political confidence. Political confidence and conspiracy mentality are positively correlated among Democrats and Republicans.

Study 2 assessed participants’ views of their preferred political party’s prospects. Political confidence was positively associated with conspiracy mentality (see Fig 3). Among candidate linear models with the difference between weighted hazard and benefit credulity as the response, and various combinations of political orientation, demographics, and confidence as predictors, confidence was a borderline insignificant negative predictor of negatively-biased credulity in the best-fitting models (see S1 Supporting information). However, when treating hazard and benefit credulity as separate responses, confidence was a strong positive predictor of benefit credulity, and a weak positive predictor of hazard credulity (see S1 Supporting information). The negative relationship between confidence and credulity bias is thus a result of the stronger positive correlation between confidence and benefit credulity.

Further, awareness of the special election, concern for the outcome of the special election, and media exposure were highly correlated with each other (*rs* > .65). We then z-scored and summed these measures to create an “engagement” variable for analysis. Among Republicans and Democrats who were aware of Doug Jones’ victory, neither party (*b* = .12, *p* = .625) nor political engagement (*b* = .09, *p* = .227) were predictive of confidence (see S1 Supporting information for full results).

## Discussion

The 2016 U.S. federal elections resulted in a dramatic inversion of the positions of the two principal political parties in the power structure, allowing us to test between two competing explanations for why, in prior work, conservatism was positively correlated with heightened credulity toward information about hazards relative to credulity toward information concerning benefits. In two studies, we robustly replicated prior results. In previously published research conducted prior to the 2016 elections [14], the authors reported effect sizes between β = .12 and β = .19 for the relationship between negatively-biased credulity and conservatism. In the current two studies—conducted after the 2016 elections—effect sizes were comparable (Study 1: β = .23, Study 2: β = .22). While both the core dependent and independent measures were included in all the above studies, caution is in order when drawing conclusions from this comparison, in part because of differences between the studies in both the other measures used, and in the representativeness of the populations sampled. However, the consistency of results both before and after the 2016 elections suggest that, reflecting an endogenous feature of a broader adaptive strategy that places a premium on addressing the possibility of future hazards, there is indeed an inherent relationship between conservatism and negatively-biased credulity. Resultantly, pre-election results are unlikely to have been the product of a power structure wherein conservatism was associated with diminished status in the political hierarchy.

Across the set of studies reported here and in Fessler et al [14], social and military conservatism are better predictors of negatively-biased credulity than is economic conservatism (see S1 Supporting information). Logically, both social and militaristic conservatism—which includes conservative views on the military, obedience to authority, and punishment—should be associated with belief in a dangerous world, in contrast to economic conservatism, which is conceptually orthogonal to exposure to hazard.

Tests of the relationship between conspiracism and political orientation largely reinforce the above findings: even though conservatives for the most part hold the reins of the federal government at the time of this research, conservatism is positively associated with conspiracism. Further, conspiracism and hazard credulity are themselves correlated, suggesting that they may share core features. Individuals who both see the world as relatively dangerous and find the possibility of future threat more likely may manifest that worldview when assessing an array of hazard-related information, including both generic conspiracy statements, and ideas having nothing to do with politics or power. Additionally, if individuals self-assort into political parties on the basis of similarity in worldview, within-group information exchange may well both heighten initial propensities in this regard and leverage that to further solidarity in opposition to the rival party. Future research might investigate these relationships more closely.

Political confidence negatively predicted negatively-biased credulity in our models.

Towards explaining this, we first note that this negative relationship is driven by a significant positive correlation between envisioned political success and benefit credulity, potentially reflecting a general predisposition toward optimism: participants who see benefits as more likely are also more confident in their preferred political party’s future success. Further, more politically confident participants displayed greater conspiracism. To the extent that confidence in the prospects of one’s preferred political party does indeed reflect the sense that one’s interests are prevailing, or will prevail, in political contests (see discussion of local versus national power structures, below), this pattern is inconsistent with the notion that conspiratorial tendencies thrive among those who consider themselves political “losers”. Additionally, among Democrats and Republicans aware of Doug Jones’ victory in the 2017 senatorial special election in Alabama, party and engagement were not predictive of confidence. This suggests that the Alabama special election likely did not substantially alter the confidence of either liberals or conservatives in the prospects of the political parties with which they respectively associated (a pattern that is understandable given the unique liabilities plaguing the losing Republican candidate in this conservative state). If so, then, although we did not measure confidence in Study 1, it may have changed little from Study 1 to Study 2, a pattern in keeping with the consistency in the associations between political orientation, negatively-biased credulity, and conspiracism across Studies 1 and 2.

According to the political hierarchy account, out-of-power groups—regardless of political orientation—are more likely to endorse conspiracy theories as a way both to increase ingroup cohesion, and to direct efforts towards recapturing political power [41], [42]. First, our results moderately challenge the view that previously documented political differences in conspiricism [38]–[40] were principally attributable to the structure of the political power hierarchy that was in place when those differences were observed, although we acknowledge that our measure of conspiratorial predisposition was not exhaustive. More to the point, however, our results directly contradict the claim that, like differences in conspiratorial thinking, political differences in negatively-biased credulity closely map onto changes in the political power hierarchy [42]. Rather, changes to the power structure in conservatives’ favor had no effect on the relationship between conservatism and negatively-biased credulity. This calls into question one of the primary assertions underlying the power structure account: that political differences in threat reactivity are in response to predominantly exogenous cues, such as electoral losses [42]. Instead, our results suggest that stable endogenous differences exist between liberals and conservatives along that fundamental dimension of threat bias. However, we acknowledge that the exact content of specific conspiracy beliefs, and the ability to track the partisan contours of that content, might shift with power changes [38], [41], [42].

Our findings are limited in a number of ways, many of which point to future research directions. First the source of information is an important determinant of whether it is believed [32]. Our dependent measure presented “statements collected from the media,” raising the possibility that participants’ beliefs about the media may have colored our results. Importantly, however, recent evidence of partisan differences in trust in the media [46] suggests that, had identifying the media as the source of the information been a key determinant, we would have found that conservativism is associated with lower credulity overall—the opposite of the patterns observed.

Second, our theory suggests that patterns of information exposure may amplify endogenous differences. Conservative politicians and media outlets can capitalize on the association between conservatism, threat reactivity, and negatively-biased credulity by promulgating warnings of danger; in turn, consumers of these messages—particularly if confined to an “information bubble”—will increase their assessment of the world as dangerous, creating the possibility of a positive feedback loop that can exaggerate baseline differences. We measured neither degree of political engagement nor patterns of media consumption, hence future research should address this question. Relatedly, by design, the instrument used here to measure negatively-biased credulity is essentially apolitical, as our goal is to explore underlying psychological correlates of political orientation, rather than belief in claims having direct political implications. We thus infer that individuals who exhibit greater overarching negatively-biased credulity will similarly be more credulous of political statements concerning hazards; future work could test for this correlation.

Third, the prior literature is inconsistent as to the observed positive relationship between conservatism and conspiratorial predisposition [32], [33] (but see [34]–[36]), with different instruments for measuring conspiracism producing conflicting results. For our purposes, the generic nature of the statements in the CMQ enhance generalizability, thus paralleling the contents of Fessler et al.’s [14] largely non-political credulity instrument. Nevertheless, it is possible that a measure employing richly contentful statements that more closely index participants’ positions regarding particular beliefs at a particular point in time might produce different results.

Fourth, if, for our participants, the local power structure looms large relative to that in Washington D.C., changes to the latter may not constitute the alterations on which our before-and-after design is based—replications focusing on state or municipal-level reversals of dominant party would address this possibility. Relatedly, although in our sample political orientation strongly aligned with party affiliation (see S1 Supporting information), perceptions of changes to the U.S. federal power structure in the past year are likely more complex than a simple reversal from one in which liberals were on top to one in which conservatives are in charge. It is possible that individuals who both perceive the world as dangerous and are inclined to see conspiratorial agents at work believe that attempts at change will founder on entrenched interests in the major political parties, or on an institutional “deep state”; such individuals may not view the 2016 elections as resulting in a true hierarchical inversion. Finally, the endorsement of conspiracism by political elites in Washington may perpetuate heightened threat sensitivity despite the change in political power. Future research should more precisely probe participants’ views regarding change or stability in the power structure, and the degree to which elite cues could drive an association between threat sensitivity and conservatism [47].

Fifth, although results supported our predictions, negatively-biased credulity accounted for a small proportion of the overall variance. As a complex behavioral trait—with heritable, environmental, and cultural inputs—many processes contribute to one’s political orientation. In light of our results, individual variation in negatively-biased credulity is likely one of a larger number of non-mutually exclusive processes shaping the political phenotype. Along those lines, future research ought to more closely investigate how negatively-biased credulity relates to broader politically-relevant constructs such as right-wing authoritarianism. Lastly, the two theories at issue here both posit universal patterns in the intersection of beliefs and politics. However, like the vast majority of prior work in this area, the current investigations were conducted only in the U.S.; it is vital that this research be replicated across multiple societies.

While the above limitations indicate that our conclusions should be considered preliminary, our work stands in contrast with literature asserting that the tendency to see dark forces at work reflects current power dynamics. Instead, our findings are consistent with the growing corpus of research indicating that differences in political orientation are in part reflective of psychological differences in information processing, critically including sensitivity to, and cognizance of, threat. Because the functional efficacy of these differing strategies depends on the intersection of the objective level of danger in the individual’s environment and the individual’s capabilities for addressing danger, neither the conservative nor the liberal approach is inherently superior. However, independent of this issue of utilitarian efficacy, our findings add to the evidence that these strategies entail differing vulnerability to disinformation. Propaganda campaigns are as old as politics, and conventional media outlets have long supported particular agendas, to the point of becoming witting or unwitting accomplices in the dissemination of bald falsehoods. However, in the run-up to the 2016 U.S. elections, an array of actors apparently worked to disseminate egregious disinformation, potentially influencing election results; similar events have also played out in recent European contests [48]–[51]. Many of these marked disinformation campaigns (distinguished in terms of their sensationalist content and demonstrable falsehoods) disproportionately targeted—and were subsequently circulated by—political conservatives [52], [53]. Our results suggest that this constituency was likely particularly susceptible to alarmist fabrications warning of nonexistent dangers. In an ironic twist, the strategy that prioritizes safety over opportunity may have unlocked the gates for malevolent manipulators in the information age.

## Supporting information

S1 Supporting information (PDF)

S1 Appendix Survey instruments for Study 1. (PDF)

S2 Appendix Survey instruments for Study 2. (PDF)
