# Supplementary material for: Electoral fortunes reverse, mindsets do not
Source: PLoS One. 2018 Dec 14;13(12):e0208653. doi: 10.1371/journal.pone.0208653 (PMC6294387; doi:10.1371/journal.pone.0208653)
Supplement: S1 Supporting information — (PDF) [file pone.0208653.s001.pdf]

SUPPLEMENTARY MATERIAL SUPPORTING: 'Electoral Fortunes Reverse, Mindsets Do Not Political Orientation, Credulity, and Conspiracism Following the 2016 U.S. Elections'

Theodore Samore[1], Daniel M. T. Fessler[1], Colin Holbrook[2,1], & Adam Maxwell Sparks[1]

[1] Department of Anthropology and Center for Behavior, Evolution, and Culture, University of California, Los Angeles

[2] Department of Cognitive and Information Sciences, University of California, Merced

Author Note

Correspondence concerning this article should be addressed to Theodore Samore, Department of Anthropology, 341 Haines Hall, University of California, Los Angeles, Los Angeles, CA 90095-1553 U.S.A. E-mail: theo.samore@gmail.com

Abstract

Keywords:

Word count:

SUPPLEMENTARY MATERIAL SUPPORTING: 'Electoral Fortunes Reverse, Mindsets Do Not Political Orientation, Credulity, and Conspiracism Following the 2016 U.S. Elections'

Contents

Abstract **2**

SUPPLEMENTARY MATERIAL SUPPORTING: 'Electoral Fortunes Reverse, Mindsets Do Not Political Orientation, Credulity, and Conspiracism Following the 2016 U.S. Elections' **3**

Overview **5**

Section 1: Supporting Details of Main Text Claims **6**

- Results . . . 6
  - Political Orientation and Credulity . . . 6
  - Political Confidence . . . 18
- Methods . . . 19
  - Claim: conservatism was associated with major party affiliation . . . 19

Section 2: Other Interesting Results **20**

- Special Election Variables . . . 24
- Hazard credulity and Benefit credulity . . . 27
- Confidence, Hazard, and Benefit . . . 29
- Conspiracy . . . 31
  - Study 1 . . . 31
  - Study 2 . . . 48
- Sex differences . . . 57

Section 3: Information about Appendix with Extensive Details of All Models **61**

References **62**

Overview

Section 1 of this supplement provides supporting details for main text claims, with a structure paralleling that of the main text.

Section 2 presents other analyses that are interesting but largely tangential from the focus of the main text.

Section 3 describes additional analyses, publically available in raw form, that provide extensive details of all models summarized and/or referenced in earlier sections.

This document and all analyses herein were generated using R (Version 3.5.1; R Core Team, 2018) and the R-packages *AICcmodavg* (Version 2.1.1; Mazerolle, 2017), *BayesFactor* (Version 0.9.12.4.2; Morey & Rouder, 2018), *bindrcpp* (Version 0.2.2; Müller, 2018), *broom* (Version 0.5.0; Robinson, 2018), *circlize* (Version 0.4.4; Z. Gu, Gu, Eils, Schlesner, & Brors, 2014), *coda* (Version 0.19.2; Plummer, Best, Cowles, & Vines, 2006), *dplyr* (Version 0.7.7; Wickham, Francois, Henry, & Müller, 2017), *forcats* (Version 0.3.0; Wickham, 2018a), *ggplot2* (Version 3.1.0; Wickham, 2009), *jpeg* (Version 0.1.8; Urbanek, 2014), *lm.beta* (Version 1.5.1; Behrendt, 2014), *Matrix* (Version 1.2.14; Bates & Maechler, 2018), *papaja* (Version 0.1.0.9842; Aust & Barth, 2018), *psych* (Version 1.8.10; Revelle, 2018), *purrr* (Version 0.2.5; Henry & Wickham, 2017), *readr* (Version 1.1.1; Wickham, Hester, & Francois, 2017), *readxl* (Version 1.1.0; Wickham & Bryan, 2018), *snakecase* (Version 0.9.2; Grosser, 2018), *stringr* (Version 1.3.1; Wickham, 2018b), *tibble* (Version 1.4.2; Müller & Wickham, 2018), *tidyr* (Version 0.8.2; Wickham & Henry, 2018), *tidyverse* (Version 1.2.1; Wickham, 2017), and *yarrr* (Version 0.1.6; Phillips, 2017).

Table 1

Results of model with overall conservatism predicting difference between weighted hazard credulity and weighted benefit credulity for Study 1

term	b	95% CIs for b	Beta	p
(Intercept)	2.00	[-0.29, 4.29]	0.00	0.09
conserv_overall	2.62	[1.54, 3.69]	0.23	0.00
sexmale	-0.12	[-1.02, 0.78]	-0.01	0.79
ethnicitywhite	-0.38	[-1.47, 0.72]	-0.03	0.50
education	0.10	[-0.17, 0.38]	0.04	0.46
age	-0.01	[-0.05, 0.02]	-0.04	0.47
income	-0.08	[-0.17, 0.02]	-0.08	0.11

Note. Model-fit statistics: adjusted R-squared = 0.04, F(6, 421) = 4.15.

Section 1: Supporting Details of Main Text Claims

Results

Political Orientation and Credulity. In main text we summarized results of linear models predicting the difference between weighted hazard credulity and weighted benefit credulity (i.e. negatively-biased credulity). The full models for Study 1 and Study 2 are displayed in Tables 1 and 2, respectively.

We claim that substituting party for conservatism score produces similar results, and showed a figure comparing raw conservatism scores by party. Republican affiliation is associated with higher weighted negatively-biased credulity in Study 1 (Table 3) and Study 2 (Table 4).

Table 2

Results of model with overall conservatism predicting difference between weighted hazard credulity and weighted benefit credulity for Study 2

term	b	95% CIs for b	Beta	p
(Intercept)	3.82	[1.4, 6.24]	0.00	0.00
conserv_overall	2.75	[1.58, 3.93]	0.22	0.00
sexmale	-1.35	[-2.39, -0.32]	-0.12	0.01
ethnicitywhite	-0.29	[-1.64, 1.06]	-0.02	0.67
education	0.03	[-0.3, 0.36]	0.01	0.86
age	-0.02	[-0.06, 0.02]	-0.04	0.37
income	-0.13	[-0.3, 0.05]	-0.07	0.16

Note. Model-fit statistics: adjusted R-squared = 0.05, F(6, 424) = 5.13.

We claim that the relationship between conservatism and negatively-biased credulity is "robust to the removal of any one credulity item, across a suite of models with alternative predictors sets (e.g. conservatism subscales), or using unweighted credulity." We now address these three claims.

Table 3

Results of model with political party predicting difference between weighted hazard credulity and weighted benefit credulity for Study 1

term	b	95% CIs for b	Beta	p
(Intercept)	0.95	[-1.3, 3.19]	0.00	0.41
partyRepublican	2.70	[1.73, 3.67]	0.28	0.00
partyTea Party	-1.91	[-10.94, 7.12]	-0.02	0.68
partyGreen	-1.64	[-6.86, 3.58]	-0.03	0.54
partyLibertarian	0.85	[-0.97, 2.67]	0.05	0.36
partynone	0.48	[-1.15, 2.11]	0.03	0.56
sexmale	-0.20	[-1.1, 0.69]	-0.02	0.66
ethnicitywhite	-0.54	[-1.64, 0.57]	-0.05	0.34
education	0.04	[-0.24, 0.31]	0.01	0.79
age	-0.01	[-0.05, 0.02]	-0.04	0.45
income	-0.09	[-0.18, 0.01]	-0.09	0.07

Note. Model-fit statistics: adjusted R-squared = 0.05, F(10, 416) = 3.47.

Item sensitivity.

Is the relationship between conservatism and credulity reported in the main text dependent on any single credulity item?

To investigate this question of item sensitivity, for each study we systematically calculate 16 different versions of the weighted credulity index, removing a different item for each version, and then fit linear models of the resulting credulity score as a function of conservatism, confidence, and demographic items. We then compare those 16 models based

Table 4

Results of model with political party predicting difference between weighted hazard credulity and weighted benefit credulity for Study 2

term	b	95% CIs for b	Beta	p
(Intercept)	2.84	[0.37, 5.31]	0.00	0.02
partyRepublican	1.67	[0.53, 2.81]	0.15	0.00
partyGreen	1.85	[-1.13, 4.83]	0.06	0.22
partyLibertarian	0.34	[-1.64, 2.32]	0.02	0.74
partyConstitutional	1.60	[-1.87, 5.07]	0.04	0.36
sexmale	-1.42	[-2.48, -0.35]	-0.13	0.01
ethnicitywhite	-0.45	[-1.84, 0.94]	-0.03	0.53
education	-0.05	[-0.39, 0.28]	-0.02	0.76
age	-0.01	[-0.05, 0.03]	-0.02	0.71
income	-0.13	[-0.32, 0.05]	-0.07	0.15

Note. Model-fit statistics: adjusted R-squared = 0.02, F(9, 420) = 2.02.

on AIC.

Below we show the AIC comparison tables, and the best and worst fitting models for each study. In all four models there is a relationship between conservatism and credulity consistent with the model presented in main text. For all 16 models from each study, see Section 3.

Table 5

Study 1. Comparison of models predicting weighted negatively-biased credulity

	predictors in model	AICc	Delta_AICc	AICcWt	Cum.Wt
6	wh_sharks	2,517.22	0.00	0.80	0.80
13	wb_cats	2,520.03	2.81	0.20	1.00
7	wh_exit_door	2,541.44	24.22	0.00	1.00
15	wb_upgrade	2,545.15	27.93	0.00	1.00
14	wb_stockwood	2,546.44	29.22	0.00	1.00
5	wh_terrorism	2,548.72	31.50	0.00	1.00
16	wb_lightning_car	2,550.10	32.89	0.00	1.00
9	wb_batteries	2,559.16	41.95	0.00	1.00
11	wb_exercise	2,560.76	43.54	0.00	1.00
1	wh_credit_cards_phone	2,561.54	44.32	0.00	1.00
10	wb_carrots	2,563.17	45.95	0.00	1.00
2	wh_kale	2,565.34	48.12	0.00	1.00
4	wh_keycards	2,567.56	50.34	0.00	1.00
3	wh_running	2,569.27	52.06	0.00	1.00
12	wb_credit_cards_credit_score	2,573.23	56.01	0.00	1.00
8	wh_lightning	2,583.04	65.82	0.00	1.00

Note. All models also include demographics. Lower AICc implies a more parsimonious model. AICcWt is interprettable as the weight of evidence for the model being the best among candidates, given the data.

Table 6

Results of model with political party predicting difference between weighted hazard credulity and weighted benefit credulity for Study 1 – best model with single item removed

term	b	95% CIs for b	Beta	p
(Intercept)	2.49	[0.22, 4.76]	0.00	0.03
conserv_overall	2.38	[1.31, 3.44]	0.21	0.00
sexmale	-0.10	[-0.99, 0.79]	-0.01	0.83
ethnicitywhite	-0.15	[-1.24, 0.93]	-0.01	0.78
education	0.04	[-0.24, 0.31]	0.01	0.79
age	-0.01	[-0.05, 0.02]	-0.03	0.51
income	-0.06	[-0.15, 0.03]	-0.06	0.22

Note. Model-fit statistics: adjusted R-squared = 0.03, F(6, 421) = 3.4.

Study 1.

AIC comparisons of the 16 models for Study 1 based on all scales with a single dropped item is shown in Table 5. The best (Table 6) and worst models (Table 7) removed the “sharks” and “lightning” items, respectively. Both show a positive relationship between negatively-biased credulity and conservatism.

Table 7

Results of model with political party predicting difference between weighted hazard credulity and weighted benefit credulity for Study 1 – worst model with single item removed

term	b	95% CIs for b	Beta	p
(Intercept)	1.74	[-0.71, 4.19]	0.00	0.16
conserv_overall	2.80	[1.65, 3.95]	0.23	0.00
sexmale	-0.01	[-0.97, 0.95]	0.00	0.98
ethnicitywhite	-0.55	[-1.72, 0.62]	-0.04	0.36
education	0.09	[-0.2, 0.38]	0.03	0.55
age	-0.01	[-0.05, 0.03]	-0.03	0.53
income	-0.06	[-0.16, 0.04]	-0.06	0.24

Note. Model-fit statistics: adjusted R-squared = 0.04, F(6, 421) = 4.01.

Study 2.

The best (Table 9) and worst models (Table 10) removed the “exit door” and “credit card phone” items, respectively. Conservatism is associated with negatively-biased credulity in both.

Unweighted credulity.

We re-ran the models presented in the main text, except that unweighted negatively-biased credulity scores are the DV instead of weighted credulity. For both studies (Study 1: Table 11, Study 2: Table 12), conservatism remains positively associated with negatively-biased credulity.

Table 8

Comparison of models predicting weighted negatively-biased credulity

predictors in model	AICc	Delta_AICc	AICcWt	Cum.Wt
wh_exit_door	2,663.52	0.00	0.86	0.86
wh_terrorism	2,668.03	4.50	0.09	0.95
wb_cats	2,670.22	6.70	0.03	0.98
wh_sharks	2,672.18	8.66	0.01	0.99
wb_upgrade	2,673.76	10.24	0.01	1.00
wb_lightning_car	2,675.45	11.93	0.00	1.00
wb_batteries	2,696.79	33.27	0.00	1.00
wh_lightning	2,698.87	35.35	0.00	1.00
wb_credit_cards_credit_score	2,702.39	38.87	0.00	1.00
wb_exercise	2,703.84	40.32	0.00	1.00
wb_stockwood	2,707.42	43.89	0.00	1.00
wh_running	2,709.86	46.33	0.00	1.00
wh_kale	2,711.13	47.61	0.00	1.00
wb_carrots	2,712.03	48.50	0.00	1.00
wh_keycards	2,713.36	49.84	0.00	1.00
wh_credit_cards_phone	2,742.28	78.76	0.00	1.00

Note. Study 2. All models also include demographics. Lower AICc implies a more parsimonious model. AICcWt is interprettable as the weight of evidence for the model being the best among candidates, given the data.

Table 9

Results of model with political party predicting difference between weighted hazard credulity and weighted benefit credulity for Study 1 – best model with single item removed

term	b	95% CIs for b	Beta	p
(Intercept)	3.80	[1.41, 6.2]	0.00	0.00
conserv_overall	2.87	[1.71, 4.03]	0.23	0.00
sexmale	-1.77	[-2.8, -0.75]	-0.16	0.00
ethnicitywhite	-0.44	[-1.77, 0.9]	-0.03	0.52
education	0.05	[-0.28, 0.38]	0.02	0.75
age	-0.02	[-0.06, 0.03]	-0.04	0.46
income	-0.14	[-0.31, 0.04]	-0.07	0.13

Note. Model-fit statistics: adjusted R-squared = 0.07, F(6, 424) = 6.48.

Alternative predictor sets.

Main text featured models similar to those used by Fessler, Pisor and Holbrook (2017). We fit a series of alternative linear models of weighted negatively-biased credulity as functions of various combinations of predictor variables (overall conservatism, conservatism subscales, party, confidence) and/or their interactions; all models include demographics as predictors. We can then use AIC to compare all models for a given outcome.

Here we show that, across a wide variety of reasonable linear models, our data support the core claim that political conservatism is associated with greater hazard-biased credulity.

Table 10

Results of model with political party predicting difference between weighted hazard credulity and weighted benefit credulity for Study 1 – worst model with single item removed

term	b	95% CIs for b	Beta	p
(Intercept)	4.26	[1.63, 6.88]	0.00	0.00
conserv_overall	2.79	[1.52, 4.06]	0.21	0.00
sexmale	-1.14	[-2.26, -0.01]	-0.09	0.05
ethnicitywhite	-0.04	[-1.51, 1.42]	0.00	0.95
education	0.05	[-0.31, 0.41]	0.01	0.79
age	-0.02	[-0.07, 0.02]	-0.05	0.34
income	-0.15	[-0.35, 0.04]	-0.08	0.12

Note. Model-fit statistics: adjusted R-squared = 0.04, F(6, 424) = 4.27.

Study 1.

AIC comparisons (Table 13) suggest that the main text featured model ("con_all") is unlikely to be the best model. However the best model (Table 14) all available models (see Section 3) are intepretable as showing that conservatism (whether overall or a subscale) and/or Republican affiliation are associated with higher levels of weighted negatively-biased credulity.

Table 11

Results of model with political party predicting difference between unweighted hazard credulity and weighted benefit credulity for Study 1

term	b	95% CIs for b	Beta	p
(Intercept)	0.25	[-0.14, 0.64]	0.00	0.21
conserv_overall	0.52	[0.34, 0.7]	0.27	0.00
sexmale	-0.02	[-0.17, 0.13]	-0.01	0.80
ethnicitywhite	-0.11	[-0.3, 0.07]	-0.06	0.23
education	0.02	[-0.03, 0.06]	0.03	0.51
age	0.00	[-0.01, 0]	-0.05	0.31
income	-0.02	[-0.03, 0]	-0.10	0.03

Note. Model-fit statistics: adjusted R-squared = 0.06, F(6, 421) = 5.81.

Study 2.

For Study 2, we first fit the same series of models as used for Study 1. After this we will consider models that also include the political confidence measure that was only used in Study 2.

AIC comparisons (Table 15) suggest that the main text featured model ("con_all") is unlikely to be the best model. However the best model (Table 16) all available models (Section 3) are intepretable as showing that conservatism (whether overall or a subscale) and/or Republican affiliation are associated with higher levels of weighted negatively-biased credulity.

Table 12

Results of model with political party predicting difference between unweighted hazard credulity and weighted benefit credulity for Study 2

term	b	95% CIs for b	Beta	p
(Intercept)	0.33	[-0.03, 0.7]	0.00	0.07
conserv_overall	0.42	[0.25, 0.6]	0.23	0.00
sexmale	-0.15	[-0.31, 0]	-0.09	0.05
ethnicitywhite	-0.20	[-0.4, 0]	-0.09	0.05
education	0.00	[-0.05, 0.05]	-0.01	0.86
age	0.00	[-0.01, 0.01]	0.01	0.85
income	0.00	[-0.03, 0.03]	0.00	0.97

Note. Model-fit statistics: adjusted R-squared = 0.06, F(6, 424) = 5.18.

Study 2: Adding confidence as a predictor.

Among the model set allowing political confidence as a predictor, AIC comparisons (Table 17) suggest that the main text featured model ("con_all") is unlikely to be the best model. However the best model (Table 18) all available models (Section 3) are intepretable as showing that conservatism (whether overall or a subscale) and/or Republican affiliation are associated with higher levels of weighted negatively-biased credulity.

Confidence is included in many of the best models, and is always a negative predictor of credulity, but typically with a borderline-insignificant standarized coefficient roughly 1/3rd the size of conservatism (Section 3). Furthermore no models including an interaction term involving confidence are among the best models.

Table 13

Study 1. Comparison of models predicting weighted negatively-biased credulity

	predictors in model	AICc	Delta_AICc	AICcWt	Cum.Wt
2	party	2,518.01	0.00	0.55	0.55
3	con_all + party	2,518.85	0.84	0.36	0.92
6	con_mil + con_econ + con_soc + party	2,522.93	4.92	0.05	0.96
1	con_all	2,524.89	6.87	0.02	0.98
4	con_all * party	2,525.97	7.96	0.01	0.99
9	con_mil	2,528.54	10.52	0.00	0.99
8	con_soc	2,528.66	10.65	0.00	1.00
5	con_mil + con_econ + con_soc	2,528.70	10.69	0.00	1.00
7	con_econ	2,535.44	17.42	0.00	1.00

Note. All models also include demographics. Lower AICc implies a more parsimonious model. AICcWt is interprettable as the weight of evidence for the model being the best among candidates, given the data.

Political Confidence. In main text we claim that confidence is a borderline-insignificant negative predictor of negatively-biased credulity in the best-fitting models. This is supported in the section above on alternative models for Study 2.

We also claim that this negative relationship is a result of confidence being a stronger positive predictor of benefit credulity than of hazard credulity. This is supported below in Section 2.

Table 14

Results of best model with political party predicting difference between unweighted hazard credulity and weighted benefit credulity for Study 1

term	b	95% CIs for b	Beta	p
(Intercept)	0.95	[-1.3, 3.19]	0.00	0.41
partyRepublican	2.70	[1.73, 3.67]	0.28	0.00
partyTea Party	-1.91	[-10.94, 7.12]	-0.02	0.68
partyGreen	-1.64	[-6.86, 3.58]	-0.03	0.54
partyLibertarian	0.85	[-0.97, 2.67]	0.05	0.36
partynone	0.48	[-1.15, 2.11]	0.03	0.56
sexmale	-0.20	[-1.1, 0.69]	-0.02	0.66
ethnicitywhite	-0.54	[-1.64, 0.57]	-0.05	0.34
education	0.04	[-0.24, 0.31]	0.01	0.79
age	-0.01	[-0.05, 0.02]	-0.04	0.45
income	-0.09	[-0.18, 0.01]	-0.09	0.07

Note. Model-fit statistics: adjusted R-squared = 0.05, F(10, 416) = 3.47.

Methods

Claim: conservatism was associated with major party affiliation. See Figure 1.

Table 15

Study 2. Comparison of models predicting weighted negatively-biased credulity

predictors in model	AICc	Delta_AICc	AICcWt	Cum.Wt
con_mil	2,665.61	0.00	0.72	0.72
con_mil + con_econ + con_soc + party	2,669.12	3.51	0.12	0.85
con_mil + con_econ + con_soc	2,669.74	4.13	0.09	0.94
con_all + party	2,671.74	6.13	0.03	0.97
con_all	2,672.34	6.73	0.02	1.00
con_all * party	2,677.13	11.52	0.00	1.00
con_econ	2,681.84	16.23	0.00	1.00
con_soc	2,681.91	16.30	0.00	1.00
party	2,684.34	18.73	0.00	1.00

Note. All models also include demographics. Lower AICc implies a more parsimonious model. AICcWt is interprettable as the weight of evidence for the model being the best among candidates, given the data.

Section 2: Other Interesting Results

In the first part of this section, we analyze weighted hazard and benefit credulity separately, as a way of breaking down the main text results that focus on difference scores. We first see that there is a consistent positive association between conservatism and hazard credulity, but this pattern is not consistent for benefits credulity. We then consider more complicated models. For either form of credulity, we find that the best models include an interaction between party and conservatism. Decomposing this interaction reveals significant positive associations between conservatism and (either type of) credulity within Democrats, but not within Republicans.

Table 16

Results of best model with political party predicting difference between unweighted hazard credulity and weighted benefit credulity for Study 2

term	b	95% CIs for b	Beta	p
(Intercept)	1.48	[-0.79, 3.74]	0.00	0.20
con_mil	2.15	[1.18, 3.12]	0.21	0.00
sexmale	0.02	[-0.88, 0.92]	0.00	0.97
ethnicitywhite	-0.27	[-1.36, 0.83]	-0.02	0.63
education	0.08	[-0.2, 0.35]	0.03	0.57
age	-0.01	[-0.05, 0.03]	-0.02	0.62
income	-0.09	[-0.18, 0.01]	-0.08	0.08

Note. Model-fit statistics: adjusted R-squared = 0.03, F(6, 421) = 3.52.

We then compare the role of political confidence in predicting hazard or benefit credulity, finding that it is positively associated with both, but more so with benefit credulity. This pattern results in the negative relationship between political confidence and negatively-biased credulity.

Next we analyze conspiracy mentality, finding that conservatism is strongly positively associated, even when accounting for political confidence.

Finally we look at sex differences in the estimated magnitudes of hazard and benefit claims. The only noteworthy finding is that, in Study 2, men rate hazards as less severe than do women.

Table 17

Study 2. Comparison of models predicting weighted negatively-biased credulity, allowing political confidence as a predictor

predictors in model	AICc	Delta_AICc	AICcWt	Cum.Wt
con_mil + confidence + party	2,663.89	0.00	0.35	0.35
con_mil + confidence	2,664.01	0.12	0.33	0.68
con_mil	2,665.61	1.72	0.15	0.83
con_mil + con_econ + con_soc + confidence + party	2,667.85	3.96	0.05	0.88
con_mil + con_econ + con_soc + confidence	2,668.18	4.29	0.04	0.92
con_mil + con_econ + con_soc + party	2,669.12	5.23	0.03	0.94
con_mil + con_econ + con_soc	2,669.74	5.85	0.02	0.96
con_all + party + confidence	2,670.52	6.63	0.01	0.97
con_all + confidence	2,671.12	7.23	0.01	0.98
con_all + party	2,671.74	7.85	0.01	0.99
con_all	2,672.34	8.45	0.01	1.00
con_all * confidence	2,673.18	9.29	0.00	1.00
con_all * party	2,677.13	13.24	0.00	1.00
con_soc + confidence	2,680.99	17.10	0.00	1.00
con_econ + confidence	2,681.13	17.24	0.00	1.00
con_econ + confidence + party	2,681.19	17.30	0.00	1.00
con_econ	2,681.84	17.95	0.00	1.00
con_soc	2,681.91	18.02	0.00	1.00
con_all * party * confidence	2,683.68	19.79	0.00	1.00
party + confidence	2,683.95	20.06	0.00	1.00
party	2,684.34	20.45	0.00	1.00
party * confidence	2,689.59	25.70	0.00	1.00
confidence	2,690.91	27.02	0.00	1.00

Note. All models also include demographics.

Table 18

Results of best model with political party predicting difference between unweighted hazard credulity and weighted benefit credulity for Study 2

term	b	95% CIs for b	Beta	p
(Intercept)	5.83	[2.74, 8.92]	0.00	0.00
con_mil	3.51	[2.05, 4.98]	0.29	0.00
confidence	-0.26	[-0.54, 0.02]	-0.09	0.07
partyRepublican	-0.42	[-1.83, 0.99]	-0.04	0.56
partyGreen	1.94	[-1.01, 4.88]	0.06	0.20
partyLibertarian	-0.88	[-2.88, 1.11]	-0.04	0.38
partyConstitutional	0.09	[-3.34, 3.53]	0.00	0.96
sexmale	-1.58	[-2.62, -0.54]	-0.14	0.00
ethnicitywhite	-0.51	[-1.86, 0.85]	-0.04	0.46
education	0.02	[-0.3, 0.35]	0.01	0.88
age	-0.02	[-0.06, 0.02]	-0.04	0.40
income	-0.15	[-0.33, 0.03]	-0.08	0.10

Note. Model-fit statistics: adjusted R-squared = 0.07, F(11, 418) = 3.99.

Table 19

won_election_check	n	awareness_mean	concern_mean	media_mean
1.00	12	3.75	3.50	2.58
2.00	372	5.09	4.32	3.22
3.00	1	6.00	4.00	3.00
5.00	1	5.00	4.00	3.00
6.00	56	1.91	1.66	1.45

Special Election Variables

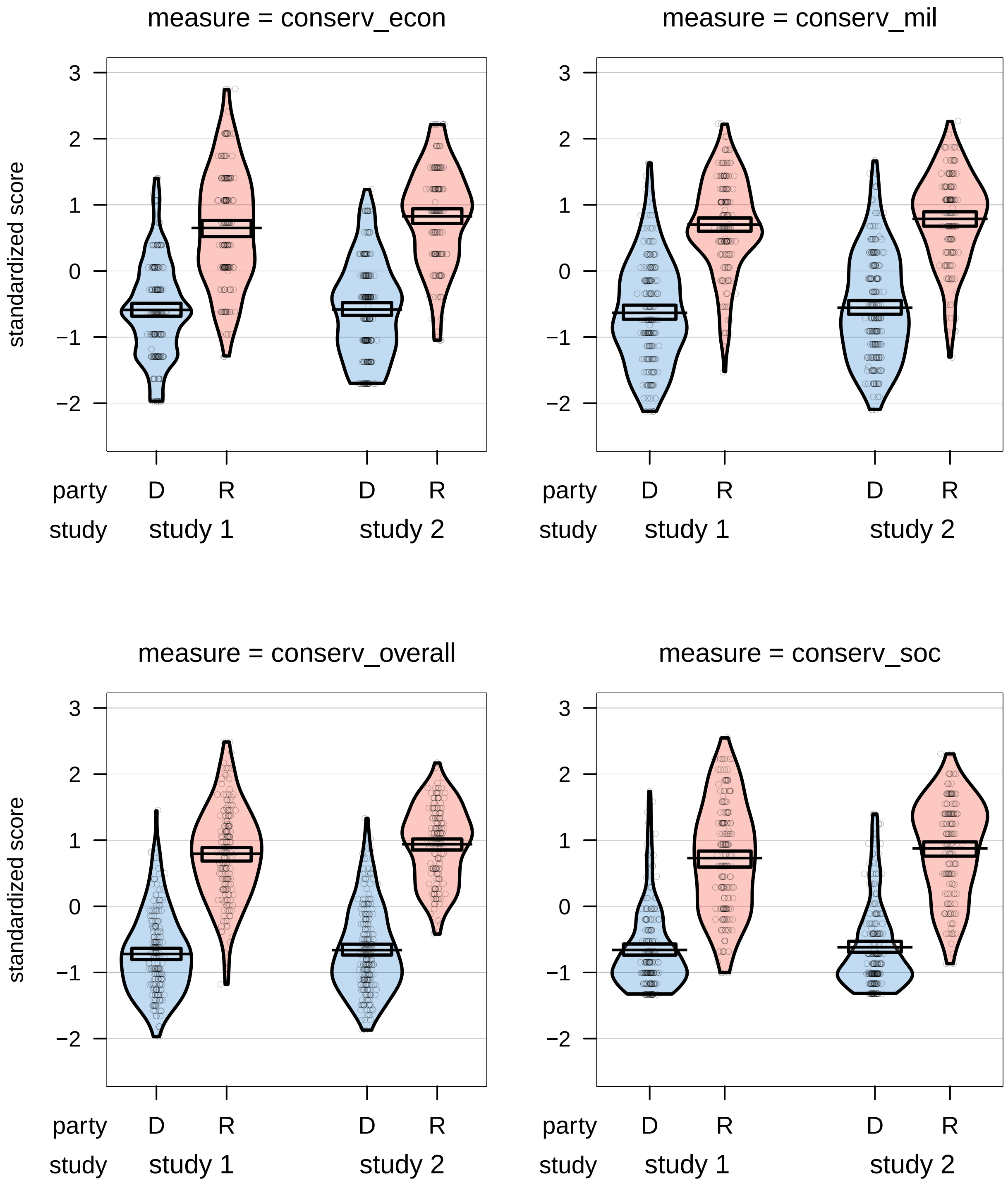

Figure 1. Conservatism as a function of major political party affiliation. Scatterplot points are individual scores, jittered along the (meaningless) horizontal axis to reduce overlap. Only data from self-identified Democrats (D) and Republicans (R) included. Beans show smoothed density of data points. Bars and boxes represent means and Bayesian 95% highest density intervals, respectively.

Table 20

	election_awareness	election_concern	election_media_attention	cred_diff_w	confidence	conserv_overal
election_awareness	1.00	0.69	0.74	-0.12	0.14	-0.32
election_concern	0.69	1.00	0.65	-0.14	0.13	-0.45
election_media_attention	0.74	0.65	1.00	-0.11	0.16	-0.25
cred_diff_w	-0.12	-0.14	-0.11	1.00	-0.08	0.22
confidence	0.14	0.13	0.16	-0.08	1.00	0.05
conserv_overall	-0.32	-0.45	-0.25	0.22	0.05	1.00

Table 21

Political engagement is equally predictive of political confidence among Republicans and Democrats who were aware of Doug Jones' victory.

Predictor	b	95% CI	$t(312)$	p
Intercept	6.97	[6.64, 7.29]	42.32	$< .001$
Engagement	0.09	[−0.06, 0.24]	1.21	.227
PartyRepublican	0.12	[−0.35, 0.58]	0.49	.625
Engagement × PartyRepublican	0.03	[−0.18, 0.25]	0.31	.756

Note. Democrats are treated as reference group, with dummy variables for Republican, and other party affiliations excluded. Model fit: ,

```
## Warning: Removed 4 rows containing non-finite values (stat_smooth).
```

```
## Warning: Removed 4 rows containing missing values (geom_point).
```

Hazard credulity and Benefit credulity

Here we analyze only weighted hazard credulity or weighted benefit credulity, rather than the difference between them, as in the main text. First, note that overall there was a positive relationship between overall conservativism and hazard credulity in Study 1 (Figure 3) and Study 2 (Figure 5), whereas conservativism predicted benefit credulity in Study 1 (Figure 7) but not Study 2 (Figure 9).

To probe predictors of credulity more thoroughly, we fit a series of models with various sets of predictors, then compare models based on AIC, as above.

For both studies and both credulity types (Tables 22 - 25), the best model involved an interaction between party and overall conservatism. To see the full details of these

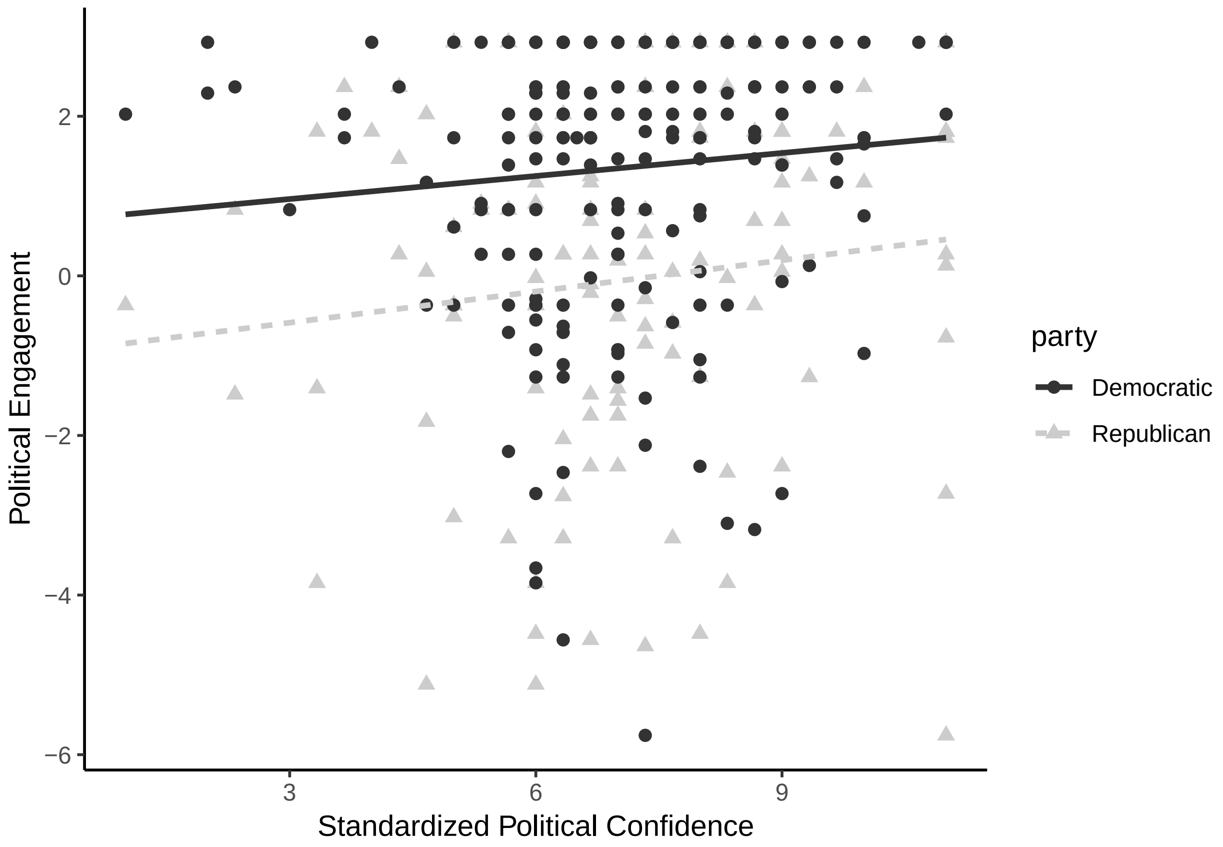

Figure 2

best-fitting models (and all lesser models), and the decomposition of the significant interaction terms see Section 3. In all cases, the interaction decomposes such that Democrats show a significant positive association between overall conservatism and credulity, whereas there is no significant relationship between those variables for Republicans. These patterns are visualized in Figures 4 - 10, which show that Republicans as a group are both more conservative and more hazard-credulous than Democrats, and that Democrats who approach Republican-level conservatism also approach Republican-level hazard credulity. There are no party differences in benefit credulity. Within party, Republicans show no association between benefit credulity and overall conservatism; Democrats show a positive relationship.

Table 22

Study 1. Comparison of models predicting weighted hazard credulity.

predictors in model	AICc	Delta_AICc	AICcWt	Cum.Wt
con_all * party	2,569.63	0.00	0.43	0.43
con_all	2,570.75	1.12	0.25	0.68
con_mil + con_econ + con_soc	2,571.96	2.33	0.14	0.82
con_all + party	2,572.82	3.18	0.09	0.90
con_soc	2,573.83	4.20	0.05	0.96
con_mil + con_econ + con_soc + party	2,574.43	4.80	0.04	1.00
con_mil	2,578.86	9.22	0.00	1.00
party	2,585.89	16.26	0.00	1.00
con_econ	2,597.06	27.42	0.00	1.00

Note. All models also include demographics. Lower AICc implies a more parsimonious model. AICcWt is interprettable as the weight of evidence for the model being the best among candidates, given the data.

Confidence, Hazard, and Benefit

In the main text we claim that confidence is a negative predictor of negatively-biased credulity because confidence predicts benefits credulity more strongly than hazard credulity. Here we support this claim.

Tables 26 & 27 compare models predicting hazard and benefit credulity, respectively. In the best model of hazard credulity (Table 28), confidence is a significant positive predictor, with a standarized coefficient of roughly .10. In the best model of benefit credulity (Table 29), confidence predicts benefit credulity with a standardized coefficent of .63, although this is qualified by a 3-way interaction term. In the second best model of benefit

Table 23

Study 2. Comparison of models predicting weighted hazard credulity.

	predictors in model	AICc	Delta_AICc	AICcWt	Cum.Wt
4	con_all * party	2,593.82	0.00	0.53	0.53
6	con_mil + con_econ + con_soc + party	2,595.66	1.84	0.21	0.74
9	con_mil	2,595.71	1.89	0.21	0.95
5	con_mil + con_econ + con_soc	2,599.03	5.21	0.04	0.99
3	con_all + party	2,601.34	7.52	0.01	1.00
1	con_all	2,606.66	12.84	0.00	1.00
8	con_soc	2,620.02	26.20	0.00	1.00
2	party	2,624.54	30.72	0.00	1.00
7	con_econ	2,627.63	33.81	0.00	1.00

Note. All models also include demographics. Lower AICc implies a more parsimonious model. AICcWt is interprettable as the weight of evidence for the model being the best among candidates, given the data.

credulity (Table 30), which is not much worse than the best model, confidence predicts benefit credulity with a standardized coefficent of .21, and this parameter is at least as large when decomposing the 3-way interaction term in the first model. Thus we conclude that confidence has a stronger relationship to benefit credulity than to hazard credulity, which drives the negative relationship between confidence and negatively-biased credulity.

Table 24

Study 1. Comparison of models predicting weighted benefit credulity.

	predictors in model	AICc	Delta_AICc	AICcWt	Cum.Wt
4	con_all * party	2,517.61	0.00	0.71	0.71
8	con_soc	2,521.49	3.89	0.10	0.81
1	con_all	2,522.36	4.75	0.07	0.87
3	con_all + party	2,522.99	5.38	0.05	0.92
9	con_mil	2,523.56	5.95	0.04	0.96
5	con_mil + con_econ + con_soc	2,524.70	7.09	0.02	0.98
6	con_mil + con_econ + con_soc + party	2,525.52	7.91	0.01	0.99
7	con_econ	2,526.86	9.25	0.01	1.00
2	party	2,530.00	12.39	0.00	1.00

Note. All models also include demographics. Lower AICc implies a more parsimonious model. AICcWt is interprettable as the weight of evidence for the model being the best among candidates, given the data.

Conspiracy

Here we analyze conspiracy mentality. First fit a series of models with various sets of predictors, then compare models based on AIC, as above.

Study 1. Model comparisons for Study 1 are displayed in Table 31. The best two models (Tables 32 & 33) shows that conservatism and hazard-biased credulity are positively associated with conspiracy mentality, as are being non-white, less educated, and older. All models are presented in Section 3.

Table 25

Study 2. Comparison of models predicting weighted benefit credulity.

predictors in model	AICc	Delta_AICc	AICcWt	Cum.Wt
con_all * party	2,584.16	0.00	0.66	0.66
party	2,587.53	3.37	0.12	0.78
con_all + party	2,588.80	4.64	0.06	0.85
con_mil	2,589.79	5.63	0.04	0.89
con_soc	2,589.96	5.80	0.04	0.92
con_all	2,590.05	5.88	0.03	0.96
con_econ	2,591.14	6.97	0.02	0.98
con_mil + con_econ + con_soc + party	2,592.10	7.94	0.01	0.99
con_mil + con_econ + con_soc	2,592.69	8.53	0.01	1.00

Note. All models also include demographics. Lower AICc implies a more parsimonious model. AICcWt is interprettable as the weight of evidence for the model being the best among candidates, given the data.

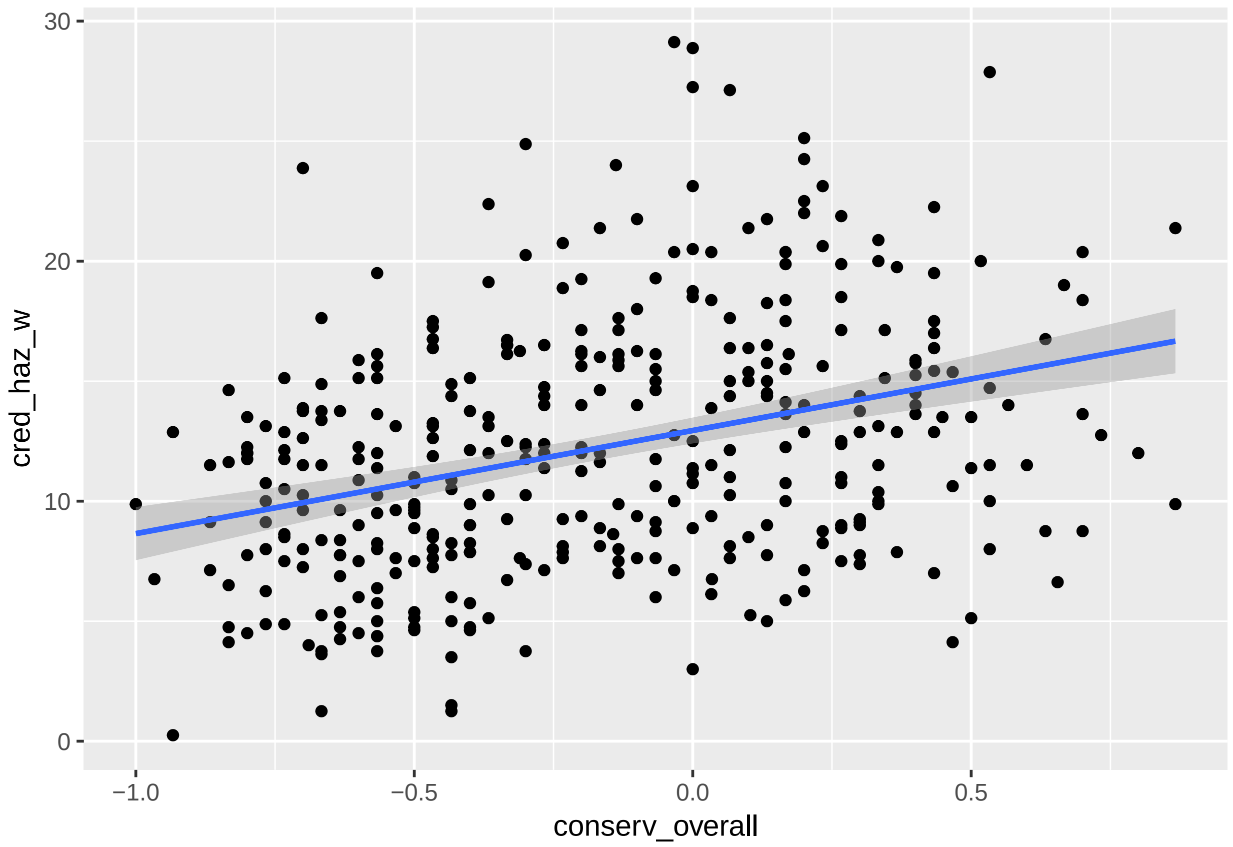

Figure 3. Relationship between conservatism and hazard credulity, Study 1

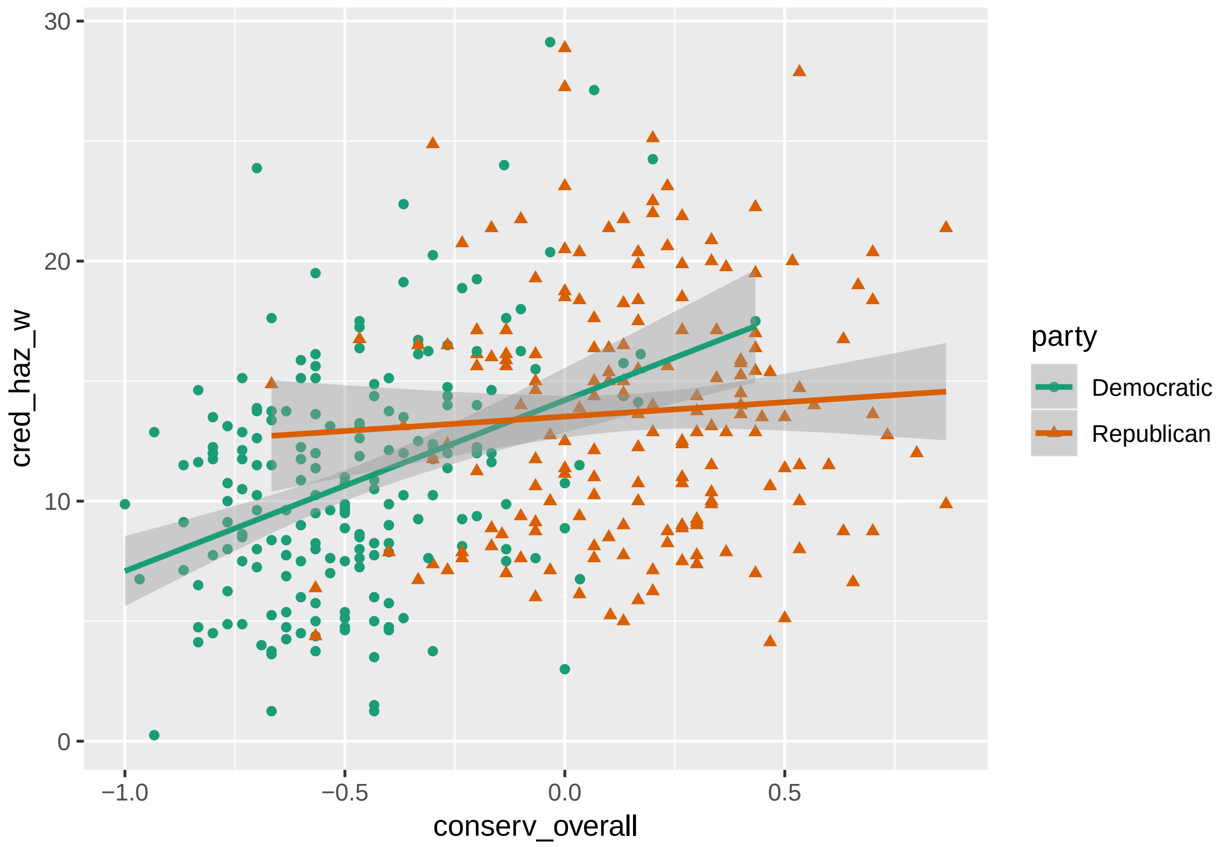

Figure 4. Relationship between conservatism and hazard credulity by party, Study 1

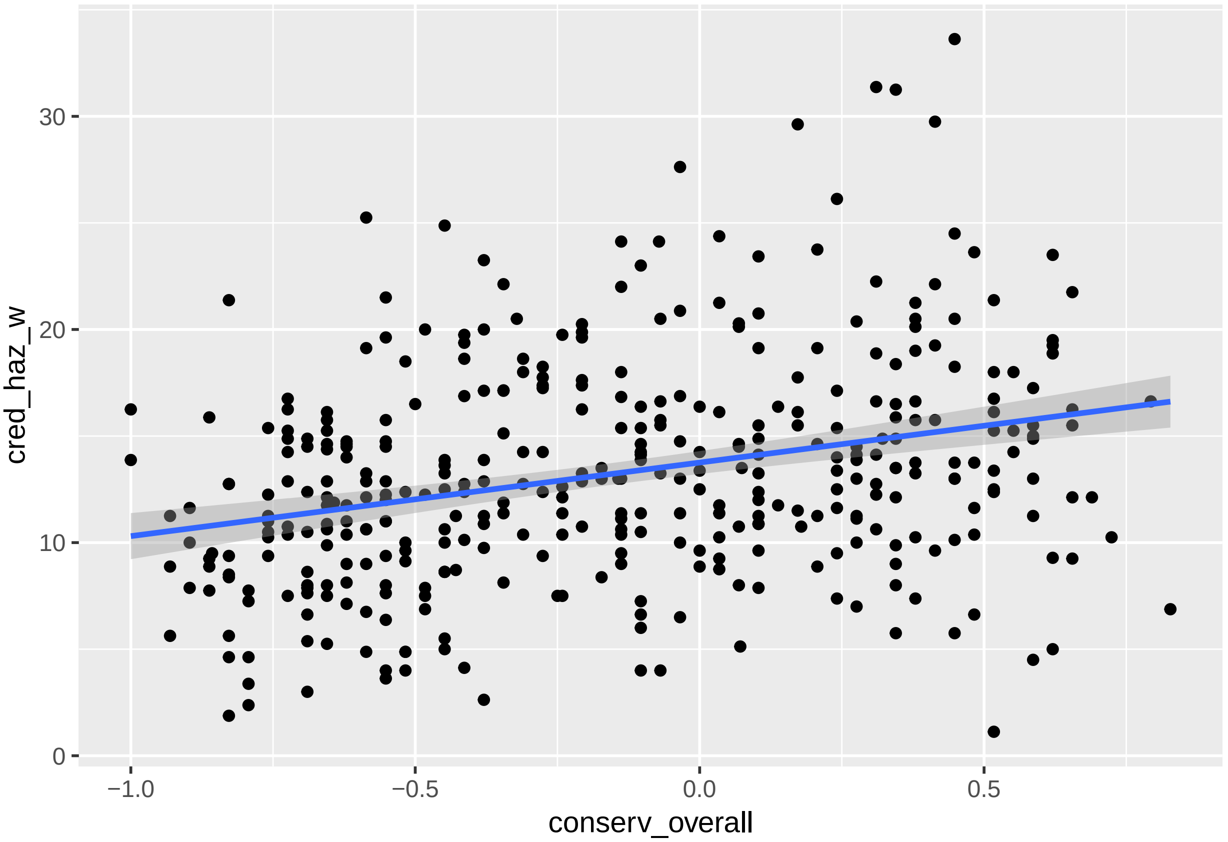

Figure 5. Relationship between conservatism and hazard credulity, Study 2

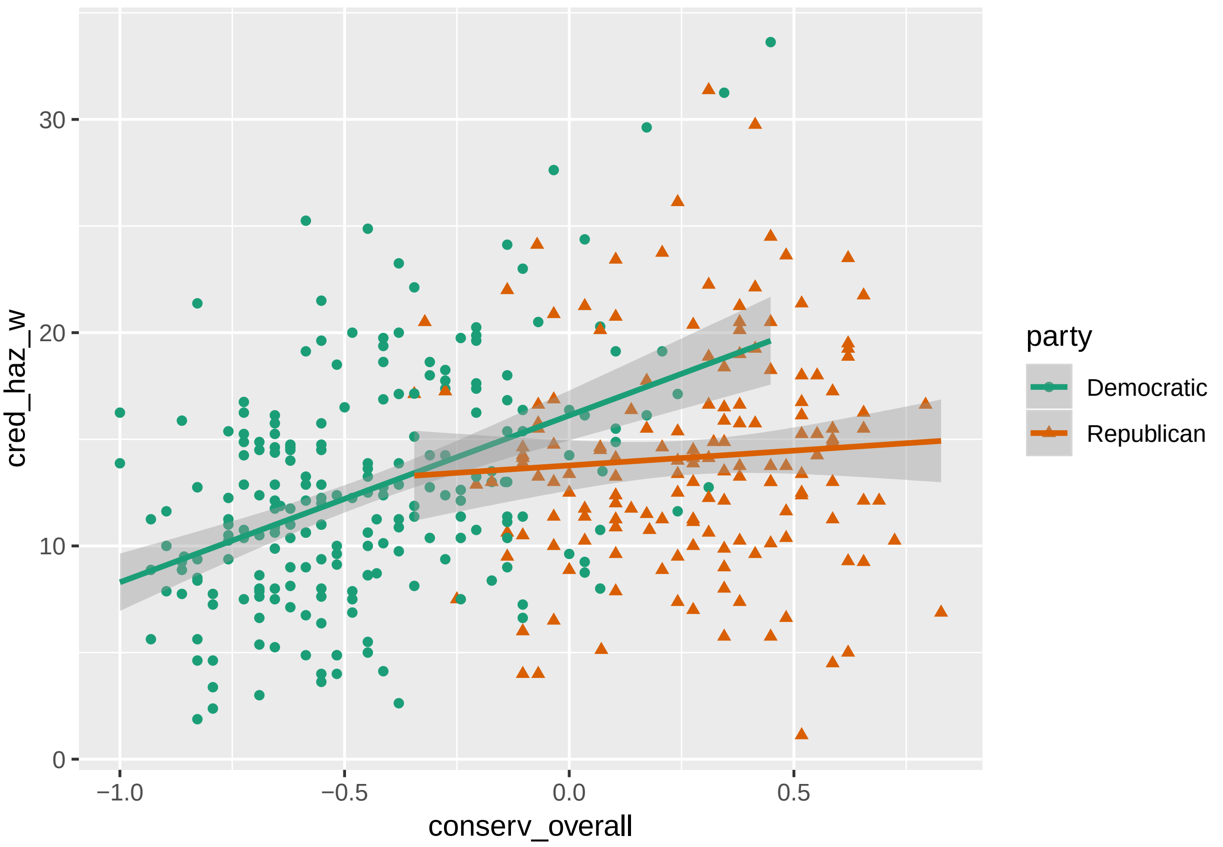

Figure 6. Relationship between conservatism and hazard credulity by party, Study 2

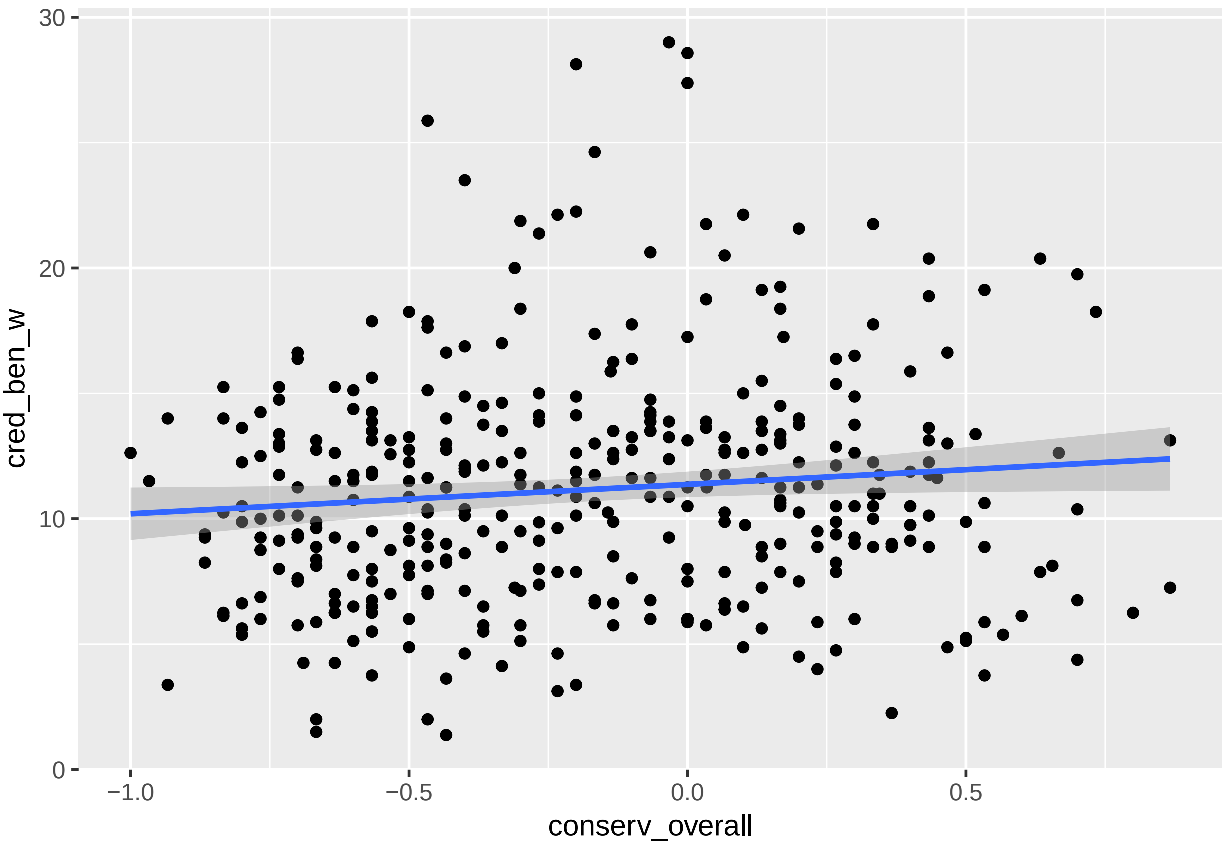

Figure 7. Relationship between conservatism and benefit credulity, Study 1

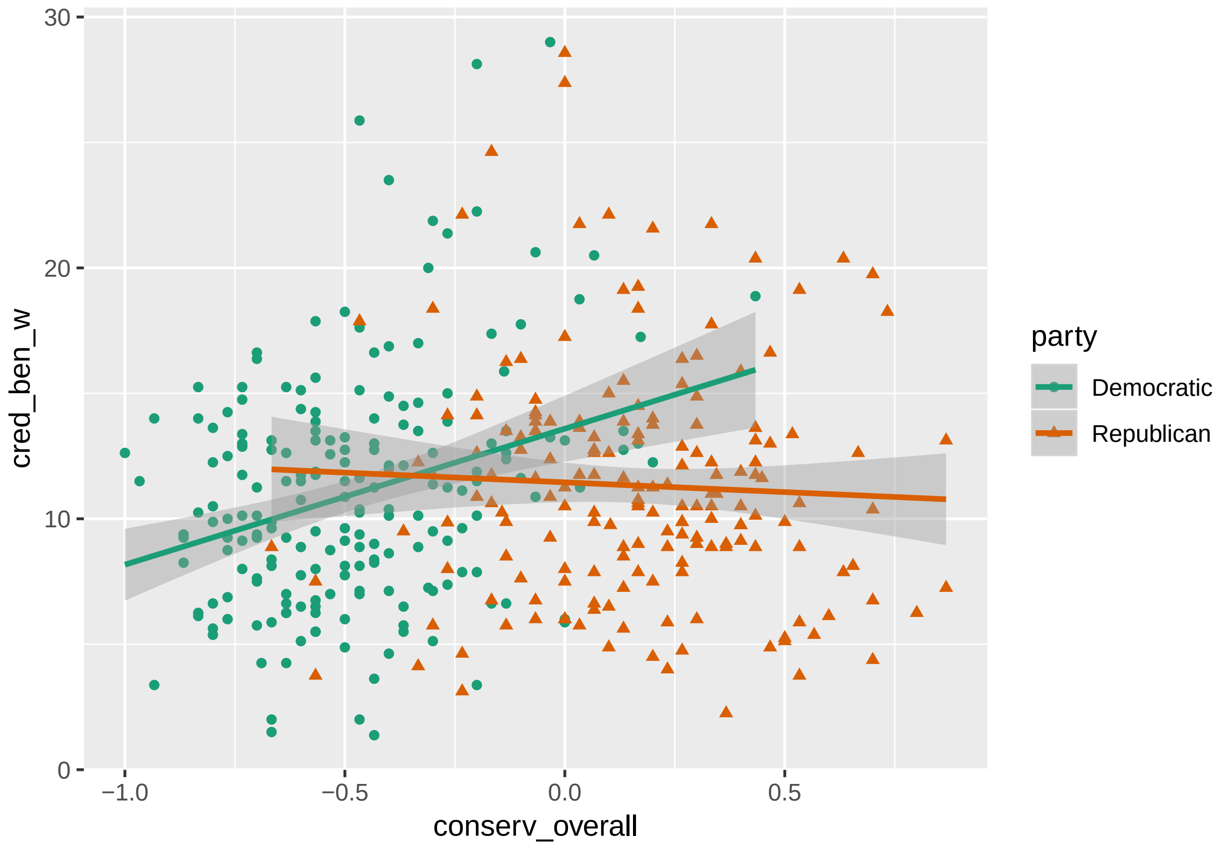

Figure 8. Relationship between conservatism and benefit credulity by party, Study 1

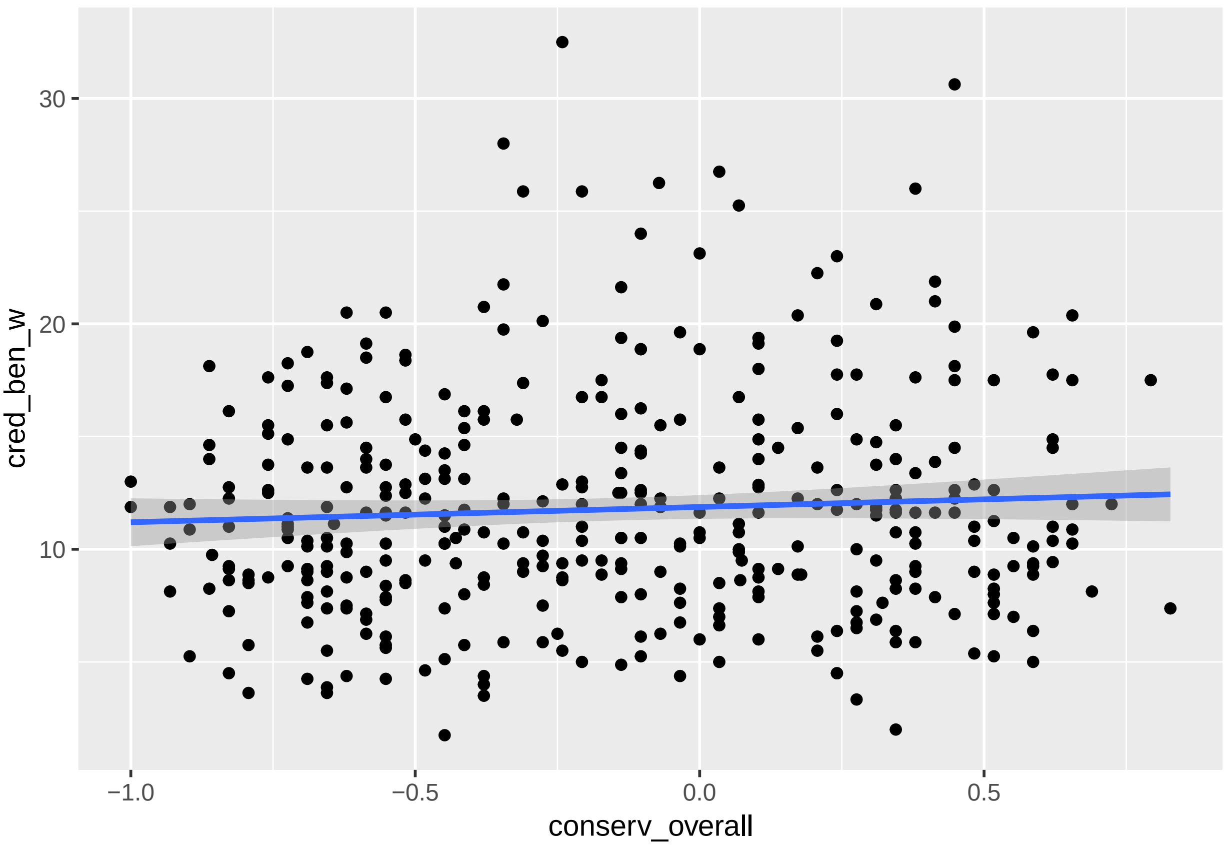

Figure 9. Relationship between conservatism and benefit credulity, Study 2

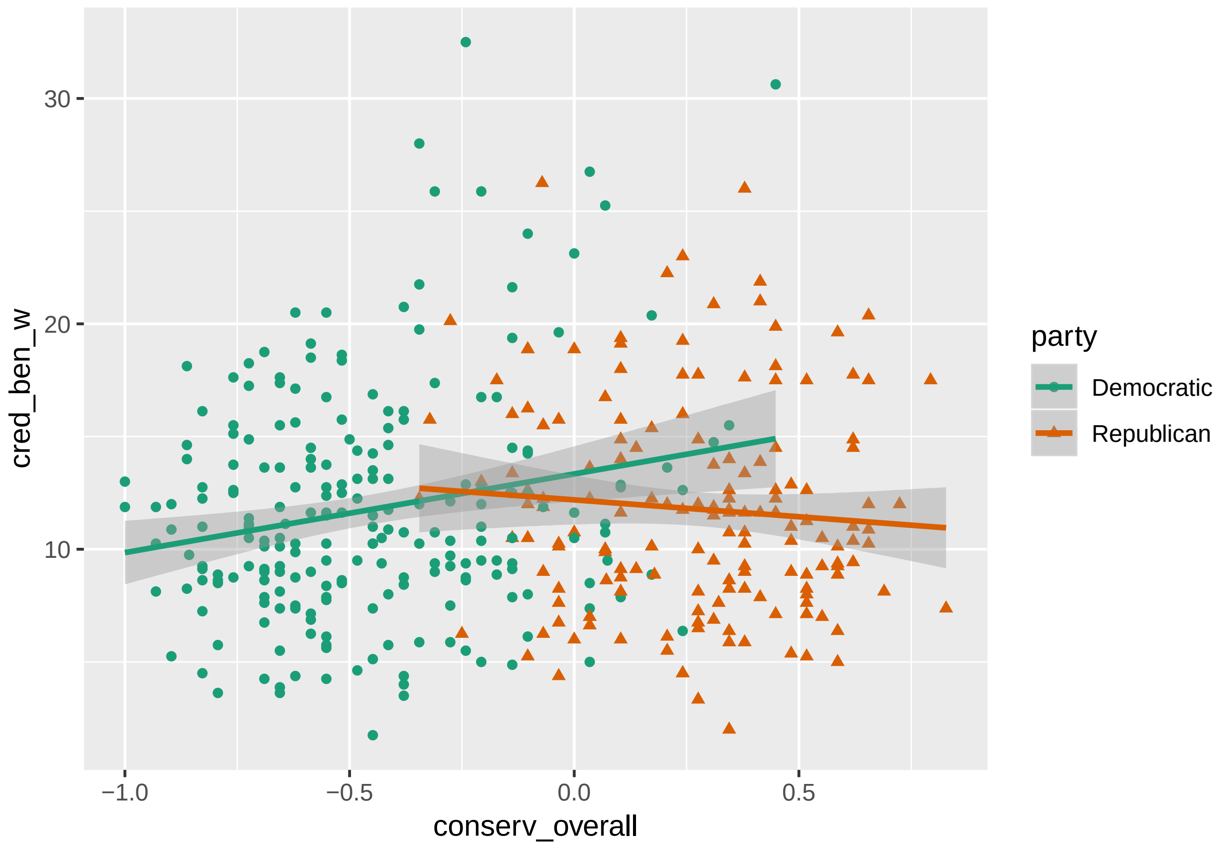

Figure 10. Relationship between conservatism and benefit credulity by party, Study 2

Table 26

Study 2. Comparison of models predicting weighted hazard credulity, allowing confidence as a predictor.

predictors in model	AICc	Delta_AICc	AICcWt	Cum.Wt
con_mil + confidence + party	2,589.86	0.00	0.55	0.55
con_mil + confidence	2,592.40	2.54	0.16	0.71
con_mil + con_econ + con_soc + confidence + party	2,593.04	3.18	0.11	0.82
con_all * party	2,593.82	3.96	0.08	0.90
con_mil + con_econ + con_soc + party	2,595.66	5.80	0.03	0.93
con_mil	2,595.71	5.85	0.03	0.96
con_mil + con_econ + con_soc + confidence	2,595.81	5.95	0.03	0.98
con_all + party + confidence	2,598.83	8.97	0.01	0.99
con_mil + con_econ + con_soc	2,599.03	9.17	0.01	1.00
con_all + party	2,601.34	11.48	0.00	1.00
con_all * party * confidence	2,602.60	12.74	0.00	1.00
con_all + confidence	2,602.98	13.12	0.00	1.00
con_all * confidence	2,605.07	15.21	0.00	1.00
con_all	2,606.66	16.80	0.00	1.00
con_soc + confidence	2,616.15	26.29	0.00	1.00
con_econ + confidence + party	2,618.96	29.10	0.00	1.00
con_soc	2,620.02	30.16	0.00	1.00
party + confidence	2,621.06	31.20	0.00	1.00
con_econ + confidence	2,623.40	33.54	0.00	1.00
party	2,624.54	34.68	0.00	1.00
con_econ	2,627.63	37.77	0.00	1.00
party * confidence	2,628.49	38.63	0.00	1.00
confidence	2,635.63	45.77	0.00	1.00

Note. All models also include demographics.

Table 27

Study 2. Comparison of models predicting weighted benefit credulity, allowing confidence as a predictor.

predictors in model	AICc	Delta_AICc	AICcWt	Cum.Wt
con_all * party * confidence	2,566.09	0.00	0.66	0.66
confidence	2,570.73	4.64	0.07	0.73
party + confidence	2,571.38	5.28	0.05	0.78
con_soc + confidence	2,571.84	5.75	0.04	0.81
con_mil + confidence	2,571.85	5.76	0.04	0.85
con_all + confidence	2,571.97	5.88	0.04	0.89
con_econ + confidence	2,572.81	6.71	0.02	0.91
con_all + party + confidence	2,573.01	6.92	0.02	0.93
con_mil + confidence + party	2,573.01	6.92	0.02	0.95
con_econ + confidence + party	2,573.39	7.29	0.02	0.97
con_all * confidence	2,574.06	7.96	0.01	0.98
con_mil + con_econ + con_soc + confidence	2,574.93	8.84	0.01	0.99
party * confidence	2,575.63	9.53	0.01	1.00
con_mil + con_econ + con_soc + confidence + party	2,576.20	10.11	0.00	1.00
con_all * party	2,584.16	18.07	0.00	1.00
party	2,587.53	21.44	0.00	1.00
con_all + party	2,588.80	22.71	0.00	1.00
con_mil	2,589.79	23.70	0.00	1.00
con_soc	2,589.96	23.87	0.00	1.00
con_all	2,590.05	23.95	0.00	1.00
con_econ	2,591.14	25.05	0.00	1.00
con_mil + con_econ + con_soc + party	2,592.10	26.01	0.00	1.00
con_mil + con_econ + con_soc	2,592.69	26.60	0.00	1.00

Note. All models also include demographics.

Table 28

Results of best model predicting hazard credulity Study 2

term	b	95% CIs for b	Beta	p
(Intercept)	14.57	[11.74, 17.4]	0.00	0.00
con_mil	3.98	[2.63, 5.32]	0.34	0.00
confidence	0.28	[0.03, 0.54]	0.10	0.03
partyRepublican	-0.56	[-1.85, 0.74]	-0.05	0.40
partyGreen	0.80	[-1.9, 3.5]	0.03	0.56
partyLibertarian	-1.08	[-2.91, 0.74]	-0.06	0.24
partyConstitutional	1.20	[-1.95, 4.35]	0.03	0.45
sexmale	-1.35	[-2.31, -0.4]	-0.13	0.01
ethnicitywhite	-1.15	[-2.4, 0.09]	-0.08	0.07
education	-0.24	[-0.54, 0.06]	-0.07	0.12
age	0.01	[-0.03, 0.05]	0.03	0.55
income	-0.23	[-0.39, -0.06]	-0.13	0.01

Note. Model-fit statistics: adjusted R-squared = 0.15, F(11, 418) = 7.72.

Table 29

Results of best model predicting benefit credulity for Study 2

term	b	95% CIs for b	Beta	p
(Intercept)	1.61	[-3.59, 6.81]	0.00	0.54
con_all	-9.51	[-19.34, 0.32]	-0.86	0.06
partyRepublican	7.52	[1.37, 13.66]	0.74	0.02
partyGreen	1.28	[-10.95, 13.5]	0.05	0.84
partyLibertarian	6.91	[-1.44, 15.26]	0.38	0.10
partyConstitutional	4.92	[-7.7, 17.54]	0.15	0.44
confidence	1.65	[0.96, 2.34]	0.63	0.00
sexmale	0.43	[-0.5, 1.36]	0.04	0.37
ethnicitywhite	-0.40	[-1.61, 0.81]	-0.03	0.51
education	-0.21	[-0.51, 0.09]	-0.07	0.17
age	0.04	[0, 0.07]	0.10	0.06
income	-0.07	[-0.23, 0.08]	-0.05	0.35
con_all:partyRepublican	7.92	[-6.08, 21.93]	0.33	0.27
con_all:partyGreen	-2.42	[-43.3, 38.46]	-0.05	0.91
con_all:partyLibertarian	-4.27	[-22.6, 14.05]	-0.09	0.65
con_all:partyConstitutional	12.18	[-35.28, 59.64]	0.13	0.61
con_all:confidence	1.86	[0.5, 3.21]	1.24	0.01
partyRepublican:confidence	-1.17	[-2.03, -0.3]	-0.86	0.01
partyGreen:confidence	-0.49	[-2.75, 1.76]	-0.10	0.67
partyLibertarian:confidence	-1.36	[-2.6, -0.11]	-0.47	0.03
partyConstitutional:confidence	-0.20	[-2.2, 1.8]	-0.04	0.84
con_all:partyRepublican:confidence	-2.07	[-4, -0.14]	-0.66	0.04
con_all:partyGreen:confidence	0.07	[-6.87, 7.01]	0.01	0.99
con_all:partyLibertarian:confidence	0.31	[-2.29, 2.92]	0.04	0.81
con_all:partyConstitutional:confidence	-4.12	[-10.36, 2.11]	-0.35	0.19

Note. Model-fit statistics: adjusted R-squared = 0.1, F(24, 405) = 2.98.

Table 30

Results of 2nd best model predicting benefit credulity for Study 2

term	b	95% CIs for b	Beta	p
(Intercept)	8.50	[5.85, 11.15]	0.00	0.00
confidence	0.56	[0.32, 0.81]	0.21	0.00
sexmale	0.21	[-0.71, 1.13]	0.02	0.65
ethnicitywhite	-0.69	[-1.89, 0.51]	-0.05	0.26
education	-0.28	[-0.57, 0.01]	-0.09	0.06
age	0.03	[0, 0.07]	0.08	0.09
income	-0.08	[-0.24, 0.07]	-0.05	0.30

Note. Model-fit statistics: adjusted R-squared = 0.06, F(6, 424) = 5.52.

Table 31

Study 1. Comparison of models predicting conspiracy mentality.

	predictors in model	AICc	Delta_AICc	AICcWt	Cum.Wt
16	con_econ + cred_diff_w	1,639.96	0.00	0.40	0.40
10	con_all + cred_diff_w	1,641.26	1.31	0.21	0.61
13	con_all * party + cred_diff_w	1,641.36	1.41	0.20	0.80
14	con_mil + con_econ + con_soc + cred_diff_w	1,642.61	2.65	0.11	0.91
17	con_soc + cred_diff_w	1,645.11	5.16	0.03	0.94
12	con_all + party + cred_diff_w	1,646.09	6.13	0.02	0.96
15	con_mil + con_econ + con_soc + party + cred_diff_w	1,647.57	7.62	0.01	0.97
1	con_all	1,647.98	8.02	0.01	0.98
4	con_all * party	1,648.07	8.11	0.01	0.98
18	con_mil + cred_diff_w	1,648.33	8.38	0.01	0.99
7	con_econ	1,648.34	8.39	0.01	0.99
5	con_mil + con_econ + con_soc	1,649.59	9.63	0.00	1.00
3	con_all + party	1,652.81	12.86	0.00	1.00
8	con_soc	1,652.95	13.00	0.00	1.00
11	party + cred_diff_w	1,653.50	13.54	0.00	1.00
6	con_mil + con_econ + con_soc + party	1,654.52	14.56	0.00	1.00

9	con_mil	1,656.71	16.75	0.00	1.00
2	party	1,661.07	21.12	0.00	1.00

Note. All models also include demographics. Lower AICc implies a more parsimonious model. AICcWt is interprettable as the weight of evidence for the model being the best among candidates, given the data.

Study 2. Model comparisons for Study 2 are displayed in Table 34. The best model (Table 35) shows that conservatism and hazard-biased credulity are positively associated with conspiracy mentality, as is being female. All models are presented in Section 3. If confidence is allowed as a predictor of conspiracy mentality, it appears in the best models (Table 36) as a positive predictor but conservatism remains a strong positive predictor (Table 37).

Table 32

Results of best model predicting conspiracy mentality for Study 1

term	b	95% CIs for b	Beta	p
(Intercept)	9.81	[9, 10.63]	0.00	0.00
con_econ	0.82	[0.44, 1.21]	0.20	0.00
cred_diff_w	0.06	[0.02, 0.09]	0.15	0.00
sexmale	-0.07	[-0.39, 0.25]	-0.02	0.67
ethnicitywhite	-0.48	[-0.86, -0.09]	-0.11	0.02
education	-0.24	[-0.34, -0.14]	-0.22	0.00
age	-0.01	[-0.03, 0]	-0.10	0.03
income	-0.01	[-0.04, 0.02]	-0.03	0.51

Note. Model-fit statistics: adjusted R-squared = 0.12, F(7, 420) = 9.48.

Table 33

Results of 2nd best model predicting conspiracy mentality for Study 1

term	b	95% CIs for b	Beta	p
(Intercept)	9.83	[9.01, 10.64]	0.00	0.00
con_all	0.81	[0.42, 1.2]	0.19	0.00
cred_diff_w	0.05	[0.02, 0.09]	0.14	0.00
sexmale	-0.05	[-0.37, 0.27]	-0.01	0.77
ethnicitywhite	-0.49	[-0.88, -0.1]	-0.11	0.01
education	-0.24	[-0.34, -0.14]	-0.22	0.00
age	-0.01	[-0.03, 0]	-0.10	0.04
income	-0.01	[-0.05, 0.02]	-0.04	0.43

Note. Model-fit statistics: adjusted R-squared = 0.12, F(7, 420) = 9.27.

Table 34

Study 2. Comparison of models predicting conspiracy mentality.

	predictors in model	AICc	Delta_AICc	AICcWt	Cum.Wt
12	con_all + party + cred_diff_w	1,808.12	0.00	0.53	0.53
15	con_mil + con_econ + con_soc + party + cred_diff_w	1,809.26	1.13	0.30	0.83
10	con_all + cred_diff_w	1,813.37	5.25	0.04	0.87
3	con_all + party	1,813.58	5.46	0.03	0.91
14	con_mil + con_econ + con_soc + cred_diff_w	1,813.87	5.75	0.03	0.94
17	con_soc + cred_diff_w	1,814.34	6.22	0.02	0.96
13	con_all * party + cred_diff_w	1,814.75	6.63	0.02	0.98
6	con_mil + con_econ + con_soc + party	1,815.82	7.70	0.01	0.99
16	con_econ + cred_diff_w	1,817.74	9.61	0.00	1.00
1	con_all	1,819.42	11.30	0.00	1.00
4	con_all * party	1,820.27	12.15	0.00	1.00
5	con_mil + con_econ + con_soc	1,821.11	12.99	0.00	1.00
8	con_soc	1,822.66	14.54	0.00	1.00
7	con_econ	1,826.27	18.15	0.00	1.00
11	party + cred_diff_w	1,826.72	18.60	0.00	1.00
18	con_mil + cred_diff_w	1,830.18	22.06	0.00	1.00

9	con_mil	1,837.17	29.05	0.00	1.00
2	party	1,837.27	29.15	0.00	1.00

Note. All models also include demographics. Lower AICc implies a more parsimonious model. AICcWt is interprettable as the weight of evidence for the model being the best among candidates, given the data.

Table 35

Results of best model predicting conspiracy mentality for Study 2, excluding confidence

term	b	95% CIs for b	Beta	p
(Intercept)	8.70	[7.76, 9.65]	0.00	0.00
con_all	1.58	[0.9, 2.27]	0.33	0.00
partyRepublican	-0.30	[-0.93, 0.33]	-0.07	0.36
partyGreen	1.01	[-0.06, 2.09]	0.09	0.06
partyLibertarian	0.67	[-0.09, 1.42]	0.09	0.08
partyConstitutional	-0.59	[-1.92, 0.75]	-0.04	0.39
cred_diff_w	0.05	[0.01, 0.08]	0.13	0.01
sexmale	-0.41	[-0.79, -0.02]	-0.09	0.04
ethnicitywhite	-0.35	[-0.85, 0.15]	-0.06	0.17
education	-0.12	[-0.25, 0]	-0.09	0.05
age	-0.01	[-0.03, 0]	-0.06	0.18
income	0.01	[-0.05, 0.08]	0.02	0.70

Note. Model-fit statistics: adjusted R-squared = 0.13, F(11, 418) = 7.01.

Table 36

Study 2. Comparison of models predicting conspiracy mentality.

	predictors in model	AICc	Delta_AICc	AICcWt	Cum.Wt
32	con_all + party + confidence + cred_diff_w	1,795.21	0.00	0.58	0.58
37	con_mil + con_econ + con_soc + confidence + party + cred_diff_w	1,796.11	0.90	0.37	0.95
40	con_econ + confidence + party + cred_diff_w	1,802.63	7.42	0.01	0.97
9	con_all + party + confidence	1,802.87	7.66	0.01	0.98
36	con_mil + con_econ + con_soc + confidence + cred_diff_w	1,805.03	9.82	0.00	0.98
14	con_mil + con_econ + con_soc + confidence + party	1,805.09	9.88	0.00	0.99
25	con_all + confidence + cred_diff_w	1,805.24	10.03	0.00	0.99
42	con_soc + confidence + cred_diff_w	1,805.78	10.57	0.00	0.99
26	con_all * confidence + cred_diff_w	1,807.06	11.85	0.00	1.00
30	con_all + party + cred_diff_w	1,808.12	12.91	0.00	1.00
39	con_econ + confidence + cred_diff_w	1,808.52	13.31	0.00	1.00
35	con_mil + con_econ + con_soc + party + cred_diff_w	1,809.26	14.05	0.00	1.00
45	con_mil + confidence + party + cred_diff_w	1,809.47	14.26	0.00	1.00
33	con_all * party * confidence + cred_diff_w	1,810.67	15.46	0.00	1.00
28	party + confidence + cred_diff_w	1,812.00	16.80	0.00	1.00
2	con_all + confidence	1,813.08	17.87	0.00	1.00

24	con_all + cred_diff_w	1,813.37	18.16	0.00	1.00
17	con_econ + confidence + party	1,813.46	18.26	0.00	1.00
7	con_all + party	1,813.58	18.37	0.00	1.00
34	con_mil + con_econ + con_soc + cred_diff_w	1,813.87	18.67	0.00	1.00
41	con_soc + cred_diff_w	1,814.34	19.13	0.00	1.00
13	con_mil + con_econ + con_soc + confidence	1,814.36	19.16	0.00	1.00
31	con_all * party + cred_diff_w	1,814.75	19.54	0.00	1.00
3	con_all * confidence	1,814.92	19.71	0.00	1.00
12	con_mil + con_econ + con_soc + party	1,815.82	20.61	0.00	1.00
19	con_soc + confidence	1,816.09	20.88	0.00	1.00
38	con_econ + cred_diff_w	1,817.74	22.53	0.00	1.00
22	con_mil + confidence + party	1,818.71	23.50	0.00	1.00
10	con_all * party * confidence	1,819.08	23.87	0.00	1.00
16	con_econ + confidence	1,819.08	23.87	0.00	1.00
1	con_all	1,819.42	24.21	0.00	1.00
29	party * confidence + cred_diff_w	1,819.85	24.64	0.00	1.00
8	con_all * party	1,820.27	25.06	0.00	1.00
11	con_mil + con_econ + con_soc	1,821.11	25.91	0.00	1.00
44	con_mil + confidence + cred_diff_w	1,822.21	27.00	0.00	1.00

18	con_soc	1,822.66	27.45	0.00	1.00
5	party + confidence	1,825.29	30.09	0.00	1.00
15	con_econ	1,826.27	31.07	0.00	1.00
27	party + cred_diff_w	1,826.72	31.51	0.00	1.00
43	con_mil + cred_diff_w	1,830.18	34.97	0.00	1.00
21	con_mil + confidence	1,831.17	35.96	0.00	1.00
6	party * confidence	1,833.02	37.81	0.00	1.00
46	confidence + cred_diff_w	1,834.11	38.90	0.00	1.00
20	con_mil	1,837.17	41.96	0.00	1.00
4	party	1,837.27	42.06	0.00	1.00
23	confidence	1,850.82	55.62	0.00	1.00

Note. All models also include demographics. Lower AICc implies a more parsimonious model. AICcWt is interprettable as the weight of evidence for the model being the best among candidates, given the data.

Sex differences

Models of negatively-biased credulity often show that men are less credulous than women, e.g. the featured model for Study 2 in the main text. This trend is consistent with arguments about sex differences in the general valuation of costs and benefits (Sparks et al, 2017).

To further examine this trend, we created scales using only the magnitude values (i.e. the weights) for hazard items ("costs") or benefit items ("benefits"). In Tables 38 - 41, we model those as a function of conservatism and demographics, finding that only cost estimates show a sex difference, and only for Study 2.

Table 37

Results of best model predicting conspiracy mentality for Study 2, allowing confidence

term	b	95% CIs for b	Beta	p
(Intercept)	7.33	[6.17, 8.5]	0.00	0.00
con_all	1.49	[0.81, 2.16]	0.31	0.00
partyRepublican	-0.23	[-0.85, 0.39]	-0.05	0.47
partyGreen	1.33	[0.26, 2.4]	0.11	0.02
partyLibertarian	0.91	[0.15, 1.66]	0.12	0.02
partyConstitutional	-0.50	[-1.81, 0.81]	-0.04	0.45
confidence	0.20	[0.1, 0.3]	0.18	0.00
cred_diff_w	0.05	[0.02, 0.09]	0.14	0.00
sexmale	-0.40	[-0.78, -0.02]	-0.09	0.04
ethnicitywhite	-0.32	[-0.81, 0.18]	-0.06	0.21
education	-0.12	[-0.24, 0]	-0.09	0.05
age	-0.01	[-0.03, 0]	-0.08	0.08
income	0.01	[-0.05, 0.08]	0.02	0.69

Note. Model-fit statistics: adjusted R-squared = 0.16, F(12, 417) = 7.87.

Table 38

Estimation of magnitude of hazard items, Study 1

term	b	95% CIs for b	Beta	p
(Intercept)	5.36	[4.77, 5.95]	0.00	0.00
conserv_overall	0.33	[0.05, 0.61]	0.12	0.02
sexmale	0.01	[-0.23, 0.24]	0.00	0.96
ethnicitywhite	-0.08	[-0.36, 0.2]	-0.03	0.57
education	-0.05	[-0.12, 0.02]	-0.07	0.14
age	-0.01	[-0.02, 0]	-0.05	0.27
income	-0.01	[-0.03, 0.02]	-0.03	0.51

Note. Model-fit statistics: adjusted R-squared = 0.01, F(6, 421) = 1.6.

Table 39

Estimation of magnitude of hazard items, Study 2

term	b	95% CIs for b	Beta	p
(Intercept)	5.08	[4.59, 5.56]	0.00	0.00
conserv_overall	0.37	[0.14, 0.61]	0.15	0.00
sexmale	-0.28	[-0.49, -0.07]	-0.13	0.01
ethnicitywhite	-0.05	[-0.32, 0.22]	-0.02	0.72
education	0.00	[-0.07, 0.07]	0.00	0.98
age	0.00	[-0.01, 0.01]	0.00	0.99
income	-0.06	[-0.09, -0.02]	-0.16	0.00

Note. Model-fit statistics: adjusted R-squared = 0.05, F(6, 424) = 5.02.

Table 40

Estimation of magnitude of benefit items, Study 1

term	b	95% CIs for b	Beta	p
(Intercept)	4.75	[4.25, 5.24]	0.00	0.00
conserv_overall	0.23	[0, 0.46]	0.09	0.05
sexmale	0.05	[-0.15, 0.24]	0.02	0.63
ethnicitywhite	-0.18	[-0.42, 0.05]	-0.07	0.13
education	-0.05	[-0.11, 0]	-0.09	0.07
age	-0.01	[-0.01, 0]	-0.07	0.17
income	0.00	[-0.02, 0.02]	-0.02	0.65

Note. Model-fit statistics: adjusted R-squared = 0.01, F(6, 421) = 1.93.

Table 41

Estimation of magnitude of benefit items, Study 2

term	b	95% CIs for b	Beta	p
(Intercept)	4.21	[3.74, 4.68]	0.00	0.00
conserv_overall	0.10	[-0.12, 0.33]	0.04	0.37
sexmale	-0.10	[-0.3, 0.1]	-0.05	0.34
ethnicitywhite	-0.27	[-0.53, 0]	-0.10	0.05
education	-0.04	[-0.11, 0.02]	-0.07	0.19
age	0.01	[0, 0.02]	0.11	0.03
income	-0.02	[-0.05, 0.01]	-0.06	0.27

Note. Model-fit statistics: adjusted R-squared = 0.02, F(6, 424) = 2.43.

Section 3: Information about Appendix with Extensive Details of All Models

Analyses described in Sections 1 and 2 often involved generating a large set of linear models. We typically presented a summary of an especially relevant model (e.g. one with the best AIC fit statistic) and then characterized whether patterns from the featured model match patterns among all similar models. Raw details of all such models are in the Appendix, publically available at [for peer review: https://osf.io/v8n6g/?view_only=aab4526f905247f0aa648ccd92ccc13a].

References

Aust, F., & Barth, M. (2018). *papaja: Create APA manuscripts with R Markdown.* Retrieved from https://github.com/crsh/papaja

Bates, D., & Maechler, M. (2018). *Matrix: Sparse and dense matrix classes and methods.* Retrieved from https://CRAN.R-project.org/package=Matrix

Behrendt, S. (2014). *Lm.beta: Add standardized regression coefficients to lm-objects.* Retrieved from https://CRAN.R-project.org/package=lm.beta

Grosser, M. (2018). *Snakecase: Convert strings into any case.* Retrieved from https://CRAN.R-project.org/package=snakecase

Gu, Z., Gu, L., Eils, R., Schlesner, M., & Brors, B. (2014). Circlize implements and enhances circular visualization in r. *Bioinformatics*, *30*(19), 2811–2812.

Henry, L., & Wickham, H. (2017). *Purrr: Functional programming tools.* Retrieved from https://CRAN.R-project.org/package=purrr

Mazerolle, M. J. (2017). *AICcmodavg: Model selection and multimodel inference based on (q)AIC(c).* Retrieved from https://cran.r-project.org/package=AICcmodavg

Morey, R. D., & Rouder, J. N. (2018). *BayesFactor: Computation of bayes factors for common designs.* Retrieved from https://CRAN.R-project.org/package=BayesFactor

Müller, K. (2018). *Bindrcpp: An 'rcpp' interface to active bindings.* Retrieved from https://CRAN.R-project.org/package=bindrcpp

Müller, K., & Wickham, H. (2018). *Tibble: Simple data frames.* Retrieved from

https://CRAN.R-project.org/package=tibble

Phillips, N. (2017). *Yarrr: A companion to the e-book "yarrr!: The pirate's guide to r"*. Retrieved from www.thepiratesguidetor.com

Plummer, M., Best, N., Cowles, K., & Vines, K. (2006). CODA: Convergence diagnosis and output analysis for mcmc. *R News*, *6*(1), 7–11. Retrieved from https://journal.r-project.org/archive/

R Core Team. (2018). *R: A language and environment for statistical computing*. Vienna, Austria: R Foundation for Statistical Computing. Retrieved from https://www.R-project.org/

Revelle, W. (2018). *Psych: Procedures for psychological, psychometric, and personality research*. Evanston, Illinois: Northwestern University. Retrieved from https://CRAN.R-project.org/package=psych

Robinson, D. (2018). *Broom: Convert statistical analysis objects into tidy data frames*. Retrieved from https://CRAN.R-project.org/package=broom

Urbanek, S. (2014). *Jpeg: Read and write jpeg images*. Retrieved from https://CRAN.R-project.org/package=jpeg

Wickham, H. (2009). *Ggplot2: Elegant graphics for data analysis*. Springer-Verlag New York. Retrieved from http://ggplot2.org

Wickham, H. (2017). *Tidyverse: Easily install and load the 'tidyverse'*. Retrieved from https://CRAN.R-project.org/package=tidyverse

Wickham, H. (2018a). *Forcats: Tools for working with categorical variables (factors)*.

Retrieved from https://CRAN.R-project.org/package=forcats

Wickham, H. (2018b). *Stringr: Simple, consistent wrappers for common string operations.* Retrieved from https://CRAN.R-project.org/package=stringr

Wickham, H., & Bryan, J. (2018). *Readxl: Read excel files.* Retrieved from https://CRAN.R-project.org/package=readxl

Wickham, H., & Henry, L. (2018). *Tidyr: Easily tidy data with 'spread()' and 'gather()' functions.* Retrieved from https://CRAN.R-project.org/package=tidyr

Wickham, H., Francois, R., Henry, L., & Müller, K. (2017). *Dplyr: A grammar of data manipulation.* Retrieved from https://CRAN.R-project.org/package=dplyr

Wickham, H., Hester, J., & Francois, R. (2017). *Readr: Read rectangular text data.* Retrieved from https://CRAN.R-project.org/package=readr
