# Supplementary material for: Electoral fortunes reverse, mindsets do not
Source: PLoS One. 2018 Dec 14;13(12):e0208653. doi: 10.1371/journal.pone.0208653 (PMC6294387; doi:10.1371/journal.pone.0208653)
Supplement: S1 Appendix — (PDF) [file pone.0208653.s002.pdf]

Please read the following information about the study before continuing.

You are asked to participate in a research study conducted by Daniel M.T. Fessler, PhD., Professor from the Anthropology Department at the University of California. You are a possible participant in this study if you are at least 18 years of age. Your participation in this study is entirely voluntary.

WHY IS THIS STUDY BEING DONE?
The purpose of this study is to investigate how people judge information presented in the media.

WHAT WILL HAPPEN IF I TAKE PART IN THIS RESEARCH STUDY?
If you volunteer to participate in this study, you will be asked to read a series of statements collected from the media, some of which are true and some of which are false; we will ask you to evaluate these statements. Then, you will answer questions about yourself, including your social opinions, age, gender, income, and political affiliation. The total length of time it takes to complete the study is approximately 10 minutes.

ARE THERE ANY POTENTIAL BENEFITS IF I PARTICIPATE?
The results may provide society with a fuller understanding about people's intuitions, and in turn, provide helpful information about human psychology. Those interested in research will be able to refer back to the author's website at http://www.sscnet.ucla.edu/anthro/faculty/fessler/ to see final results.

WILL I BE PAID FOR MY PARTICIPATION IN THE STUDY?
You will receive £1.00 ($1.25) in compensation for your participation in this study.

WILL INFORMATION ABOUT ME AND MY PARTICIPATION BE KEPT CONFIDENTIAL?
IP addresses will be collected and used to identify repeat participants, then deleted.

WHAT ARE MY RIGHTS IF I TAKE PART IN THE STUDY?
You can choose whether or not you want to be in the study. If you volunteer to be in the study, you may leave the study at any time without consequences by closing your web browser.

WHO CAN ANSWER QUESTIONS THAT I MIGHT HAVE ABOUT THIS STUDY?
If you have any questions, comments or concern about the study, you can contact:
Daniel M.T. Fessler, Professor of Anthropology Department of Anthropology, UCLA Los Angeles, CA 90095-1553 Email: dfessler@anthro.ucla.edu Phone: (310) 794-9252

If you want to ask questions about your rights as a research participant, or want to voice any problems or concerns you may have about the study to someone other than the researchers, please call the Office of the Human Research Protection Program (OHRPP) at 825-7122 or send a letter to OHRPP, UCLA, 11000 Kinross Avenue, Suite 102, Box 951694, Los Angeles, CA 90095-1694. Completing the survey indicates that you have agreed to participate in the study.

By continuing to the study, you state that you understand the procedures described above, that your questions have been answered to your satisfaction, and that you agree to participate.

On the next page, you will find a series of statements collected from the media.

Some of these statements are TRUE, and some of them are FALSE.

For each of the statements, please indicate, by checking the appropriate box, how confident you are that the statement is true or false. For each of the statements, you will also be asked to indicate how RISKY or BENEFICIAL you think the things described in the statement are.

Please note that your answers to each of these two questions should be thought about independently of each other. For example, you might decide that you're absolutely certain that a statement is true, but also feel that the risk or benefit described in the statement is small.

Storing batteries in a refrigerator or freezer will improve their performance.

I'm absolutely certain this statement is FALSE						*I'm absolutely certain this statement is TRUE*
○	○	○	○	○	○	○

The benefit described in this statement is SMALL						*The benefit described in this statement is LARGE*
○	○	○	○	○	○	○

Cell phones damage credit card magnetic strips, making them unusable.

	I'm absolutely certain this statement is FALSE						*I'm absolutely certain this statement is TRUE*
.	○	○	○	○	○	○	○

	The risk described in this statement is SMALL						*The risk described in this statement is LARGE*
.	○	○	○	○	○	○	○

Eating carrots results in significantly improved vision.

	I'm absolutely certain this statement is FALSE						*I'm absolutely certain this statement is TRUE*
.	○	○	○	○	○	○	○

	The benefit described in this statement is SMALL						*The benefit described in this statement is LARGE*
.	○	○	○	○	○	○	○

Kale contains thallium, a toxic heavy metal, that the plant absorbs from soil.

	I'm absolutely certain this statement is FALSE						*I'm absolutely certain this statement is TRUE*
.	○	○	○	○	○	○	○

	The risk described in this statement is SMALL						*The risk described in this statement is LARGE*
.	○	○	○	○	○	○	○

Exercising on an empty stomach burns more calories.

	I'm absolutely certain this statement is FALSE						*I'm absolutely certain this statement is TRUE*
.	○	○	○	○	○	○	○

	The benefit described in this statement is SMALL						*The benefit described in this statement is LARGE*
.	○	○	○	○	○	○	○

Long-distance running causes osteoarthritis of the knees.

	I'm absolutely certain this statement is FALSE						I'm absolutely certain this statement is TRUE
.	◯	◯	◯	◯	◯	◯	◯

	The risk described in this statement is SMALL						The risk described in this statement is LARGE
.	◯	◯	◯	◯	◯	◯	◯

Selecting credit cards that have a low credit limit improves one's credit score.

	I'm absolutely certain this statement is FALSE						*I'm absolutely certain this statement is TRUE*
.	○	○	○	○	○	○	○

	The benefit described in this statement is SMALL						*The benefit described in this statement is LARGE*
.	○	○	○	○	○	○	○

Hotel room keycards are often encoded with personal information that can be read by thieves.

	I'm absolutely certain this statement is FALSE						*I'm absolutely certain this statement is TRUE*
.	○	○	○	○	○	○	○

	The risk described in this statement is SMALL						*The risk described in this statement is LARGE*
.	○	○	○	○	○	○	○

People who own cats live longer than people who don’t.

	I'm absolutely certain this statement is FALSE						*I'm absolutely certain this statement is TRUE*
.	○	○	○	○	○	○	○

	The benefit described in this statement is SMALL						*The benefit described in this statement is LARGE*
.	○	○	○	○	○	○	○

Stockwood, California is one of the safest cities in the U.S.

	I'm absolutely certain this statement is FALSE						*I'm absolutely certain this statement is TRUE*
.	○	○	○	○	○	○	○

	The benefit described in this statement is SMALL						*The benefit described in this statement is LARGE*
.	○	○	○	○	○	○	○

Terrorist attacks in the U.S. have increased since Sept 11, 2001.

	I'm absolutely certain this statement is FALSE						*I'm absolutely certain this statement is TRUE*
.	○	○	○	○	○	○	○

	The risk described in this statement is SMALL						*The risk described in this statement is LARGE*
.	○	○	○	○	○	○	○

Sharks pose a significant risk to beachgoers.

	I'm absolutely certain this statement is FALSE						*I'm absolutely certain this statement is TRUE*
.	○	○	○	○	○	○	○

	The risk described in this statement is SMALL						*The risk described in this statement is LARGE*
.	○	○	○	○	○	○	○

When flying on major airlines, you are more likely to be upgraded from economy to business class if you ask at the gate.

	I'm absolutely certain this statement is FALSE						*I'm absolutely certain this statement is TRUE*
.	○	○	○	○	○	○	○

	The benefit described in this statement is SMALL						*The benefit described in this statement is LARGE*
.	○	○	○	○	○	○	○

In a thunderstorm, a hard-topped car can offer protection from lightning, as long as the occupants do not touch metal inside the car.

	I'm absolutely certain this statement is FALSE						*I'm absolutely certain this statement is TRUE*
.	○	○	○	○	○	○	○

	The benefit described in this statement is SMALL						*The benefit described in this statement is LARGE*
.	○	○	○	○	○	○	○

An intoxicated passenger could partially open the exit door on a commercial jetliner, causing the cabin to depressurize and the oxygen masks to deploy.

.	*I'm absolutely certain this statement is FALSE*						*I'm absolutely certain this statement is TRUE*
	○	○	○	○	○	○	○

.	*The risk described in this statement is SMALL*						*The risk described in this statement is LARGE*
	○	○	○	○	○	○	○

In the U.S., an average of 32 people are killed by lightning each year.

	I'm absolutely certain this statement is FALSE						*I'm absolutely certain this statement is TRUE*
.	○	○	○	○	○	○	○

	The risk described in this statement is SMALL						*The risk described in this statement is LARGE*
.	○	○	○	○	○	○	○

On the next page, you will find a series of statements asking about what your general beliefs. You will be asked to indicate you likely you think that each statement is true, from certainly not true, to certainly true.

I think that....

	certainly not	*extremely unlikely*	*very unlikely*	*unlikely*	*somewhat unlikely*	*undecided*	*somewhat likely*	*likely*	*very likely*	*extremely likely*	*certain*
… many very important things happen in the world, which the public is never informed about.	○	○	○	○	○	○	○	○	○	○	○
… politicians usually do not tell us the true motives for their decisions.	○	○	○	○	○	○	○	○	○	○	○
… government agencies closely monitor all citizens.	○	○	○	○	○	○	○	○	○	○	○
… events which superficially seem to lack a connection are often the result of secret activities.	○	○	○	○	○	○	○	○	○	○	○
… there are secret organizations that greatly influence political decisions.	○	○	○	○	○	○	○	○	○	○	○

Do you consider yourself American?

Is English your first language?

How would you rate your overall political orientation?

	Extremely Liberal				Moderate				Extremely Conservative
	○	○	○	○	○	○	○	○	○

Please select the term that best describes your political affiliation:

Other (please specify)

Your ethnicity:

Please indicate whether you agree or disagree, or are uncertain, with regard to each topic listed below:

	Agree	*Disagree*	*Uncertain*
**School prayer:**	○	○	○
**Pacifism:**	○	○	○
**Socialism:**	○	○	○
**Pornography:**	○	○	○
**Illegal immigration:**	○	○	○
**Women's equality:**	○	○	○
**Death penalty:**	○	○	○
**Use nuclear weapons against threats to the US :**	○	○	○
**Premarital sex:**	○	○	○
**Gay marriage:**	○	○	○
**Abortion rights:**	○	○	○
**Evolution:**	○	○	○
**Patriotism:**	○	○	○
**Biblical truth:**	○	○	○

Please indicate whether you agree or disagree, or are uncertain, with regard to each topic listed below:

	Agree	*Disagree*	*Uncertain*
**Bomb cities controlled by terrorists:**	○	○	○
**Welfare spending:**	○	○	○
**Tax cuts:**	○	○	○
**Waterboarding terror suspects:**	○	○	○
**Gun control:**	○	○	○
**Military spending:**	○	○	○
**Warrantless searches:**	○	○	○
**Globalization:**	○	○	○
**Pollution control:**	○	○	○
**Small government:**	○	○	○
**Charter schools:**	○	○	○
**Foreign aid:**	○	○	○
**Free trade:**	○	○	○
**Drone strikes**	○	○	○
**Obedience to authorities:**	○	○	○
**Compromise with enemies:**	○	○	○

Annual household income:

Education:

What is your height, to the nearest half-inch?

	Feet	Inches	Half-Inch
Height			

What is your Prolific Academy ID?

How many letters are in the word "obligatory"?

In all honesty, did you pay close attention during this study? (Please answer honestly -- your answer will improve the quality of the study, but will not affect your Prolific Academy rating in any way.)

- Yes, I paid attention throughout
- Somewhat -- I paid attention at some points but became distracted at other points
- No, I did not really pay close attention

Thank you for participating!

Your Prolific Academy Completion URL is:

https://prolific.ac/submissions/complete?cc=SATWFF85

(IMPORTANT! You must click on the link to complete the study in Prolific Academy and receive payment.)

This study is intended to investigate how social attitudes and abstract reasoning affect the evaluation of information. Please keep in mind that, although some of the statements that you evaluated are true, many of them are false.

If you would like to be informed via email as to which of the statements are true and which are false, please enter your email address in the box below, and we will email you after this project has been completed; your email address will be stored separately from your responses to all questions on this survey. After the requested information is sent, all email addresses will be deleted.

(TOTALLY OPTIONAL) Your email address:

Thank you again, very much, for your assistance. If you have any questions or concerns about this research, please feel free to contact me.

Daniel M.T. Fessler, Ph.D, Professor
Dept. of Anthropology, UCLA
Los Angeles, CA 90095-1553
Email: dfessler@anthro.ucla.edu
Tel. (310) 794-9252

You can also contact the OHRPP (formerly OPRS), Office of Human Research Protection Program, UCLA, 11000 Kinross Avenue, Suite 102, Box 951694, Los Angeles, CA 90095-1694, (310) 825-7122.
