# Supplementary material for: Electoral fortunes reverse, mindsets do not
Source: PLoS One. 2018 Dec 14;13(12):e0208653. doi: 10.1371/journal.pone.0208653 (PMC6294387; doi:10.1371/journal.pone.0208653)
Supplement: S2 Appendix — (PDF) [file pone.0208653.s003.pdf]

consent

You are asked to participate in a research study conducted by Daniel M.T. Fessler, PhD., Professor from the Anthropology Department at the University of California. You are a possible participant in this study if you are at least 18 years of age. Your participation in this study is entirely voluntary.

WHY IS THIS STUDY BEING DONE?
The purpose of this study is to investigate how people judge information presented in the media.

WHAT WILL HAPPEN IF I TAKE PART IN THIS RESEARCH STUDY?
If you volunteer to participate in this study, you will be asked to read a series of statements collected from the media, some of which are true and some of which are false; we will ask you to evaluate these statements. Then, you will answer questions about yourself, including your social opinions, age, gender, income, and political affiliation. The total length of time it takes to complete the study is approximately 10 minutes.

POTENTIAL RISKS AND DISCOMFORTS
There are no anticipated risks, but you may perceive the video as unpleasant. You are free to exit the study without consequences of any kind.

ARE THERE ANY POTENTIAL BENEFITS IF I PARTICIPATE?
The results may provide society with a fuller understanding about people's intuitions, and in turn, provide helpful information about human psychology. Those interested in research will be able to refer back to the author’s website at http://www.sscnet.ucla.edu/anthro/faculty/fessler/ to see final results.

WILL I BE PAID FOR MY PARTICIPATION IN THE STUDY?
You will receive $0.80 in compensation for your participation in this study.

WILL INFORMATION ABOUT ME AND MY PARTICIPATION BE KEPT CONFIDENTIAL?
IP addresses will be collected and used to identify repeat participants, then deleted.

WHAT ARE MY RIGHTS IF I TAKE PART IN THE STUDY?
You can choose whether or not you want to be in the study. If you volunteer to be in the study, you may leave the study at any time without consequences by closing your web browser.

WHO CAN ANSWER QUESTIONS THAT I MIGHT HAVE ABOUT THIS STUDY?
If you have any questions, comments or concern about the study, you can contact:
Daniel M.T. Fessler, Professor of Anthropology Department of Anthropology, UCLA Los Angeles, CA 90095-1553 Email: dfessler@anthro.ucla.edu Phone: (310) 794-9252

If you want to ask questions about your rights as a research participant, or want to voice any problems or concerns you may have about the study to someone other than the researchers, please call the Office of the Human Research Protection Program (OHRPP) at 825-7122 or send a letter to OHRPP, UCLA, 10889 Wilshire Blvd, Suite 830
Los Angeles, CA 90095-1406. Completing the survey indicates that you have agreed to participate in the study.

credulity intro

On the next page, you will find a series of statements collected from the media.

Some of these statements are TRUE, and some of them are FALSE.

For each of the statements, please indicate, by checking the appropriate box, how confident you are that the statement is true or false. For each of the statements, you will also be asked to indicate how RISKY or BENEFICIAL you think the things described in the statement are.

Please note that your answers to each of these two questions should be thought about independently of

each other. For example, you might decide that you’re absolutely certain that a statement is true, but also feel that the risk or benefit described in the statement is small.

cb- batteries

Storing batteries in a refrigerator or freezer will improve their performance.

I'm absolutely certain this statement is FALSE	.	.	.	.	.	*I'm absolutely certain this statement is TRUE*
○	○	○	○	○	○	○

Click to write the question text

The benefit described in this statement is SMALL	.	.	.	.	.	*The benefit described in this statement is LARGE*
○	○	○	○	○	○	○

ch- credit cards

Cell phones damage credit card magnetic strips, making them unusable.

I'm absolutely certain this statement is FALSE	2	2	2	2	2	*I'm absolutely certain this statement is TRUE*
○	○	○	○	○	○	○

Click to write the question text

The risk described in this statement is SMALL	2	2	2	2	2	*The risk described in this statement is LARGE*
○	○	○	○	○	○	○

cb- carrots

Eating carrots results in significantly improved vision.

I'm absolutely certain this statement is FALSE	2	3	4	5	6	*I'm absolutely certain this statement is TRUE*
○	○	○	○	○	○	○

Click to write the question text

The benefit described in this statement is SMALL	2	2	2	2	2	The benefit described in this statement is LARGE
○	○	○	○	○	○	○

ch- kale

Kale contains thallium, a toxic heavy metal, that the plant absorbs from soil.

I'm absolutely certain this statement is FALSE	2	2	2	2	2	I'm absolutely certain this statement is TRUE
○	○	○	○	○	○	○

Click to write the question text

The risk described in this statement is SMALL	2	2	2	2	2	The risk described in this statement is LARGE
○	○	○	○	○	○	○

cb- exercise

Exercising on an empty stomach burns more calories.

I'm absolutely certain this statement is FALSE	2	2	2	2	2	I'm absolutely certain this statement is TRUE
○	○	○	○	○	○	○

Click to write the question text

The benefit described in this statement is SMALL	2	2	2	2	2	The benefit described in this statement is LARGE
○	○	○	○	○	○	○

ch- long distance running

Long-distance running causes osteoarthritis of the knees.

I'm absolutely certain this statement is FALSE	2	2	2	2	2	I'm absolutely certain this statement is TRUE

I'm absolutely certain this statement is FALSE	2	2	2	2	2	*I'm absolutely certain this statement is TRUE*
○	○	○	○	○	○	○

Click to write the question text

The risk described in this statement is SMALL	2	2	2	2	2	*The risk described in this statement is LARGE*
○	○	○	○	○	○	○

cb- credit score

Selecting cred cards that have a low credit limit improves one's credit score.

I'm absolutely certain this statement is FALSE	2	2	2	2	2	*I'm absolutely certain this statement is TRUE*
○	○	○	○	○	○	○

Click to write the question text

The benefit described in this statement is SMALL	2	2	2	2	2	*The benefit described in this statement is LARGE*
○	○	○	○	○	○	○

ch- keycards

Hotel room keycards are often encoded with personal information that can be read by thieves.

I'm absolutely certain this statement is FALSE	2	2	2	2	2	*I'm absolutely certain this statement is TRUE*
○	○	○	○	○	○	○

Click to write the question text

The risk described in this statement is SMALL	2	2	2	2	2	*The risk described in this statement is LARGE*
○	○	○	○	○	○	○

cb- cats

People who own cats live longer than people who don't.

	I'm absolutely certain this statement is FALSE	2	2	2	2	2	I'm absolutely certain this statement is TRUE
	○	○	○	○	○	○	○

Click to write the question text

	The benefit described in this statement is SMALL	2	2	2	2	2	The benefit described in this statement is LARGE
	○	○	○	○	○	○	○

cb- stockwood

Stockwood, California is one of the safest cities in the U.S.

	I'm absolutely certain this statement is FALSE	2	2	2	2	2	I'm absolutely certain this statement is TRUE
	○	○	○	○	○	○	○

Click to write the question text

	The benefit described in this statement is SMALL	2	2	2	2	2	The benefit described in this statement is LARGE
	○	○	○	○	○	○	○

ch- terrorism

Terrorist attacks in the U.S. have increased since Sept 11, 2001.

	I'm absolutely certain this statement is FALSE	2	2	2	2	2	I'm absolutely certain this statement is TRUE
	○	○	○	○	○	○	○

Click to write the question text

	The risk described in this statement is SMALL	2	2	2	2	2	The risk described in this statement is LARGE

The risk described in this statement is SMALL	2	2	2	2	2	*The risk described in this statement is LARGE*
○	○	○	○	○	○	○

ch- sharks

Sharks pose a significant risk to beachgoers.

I'm absolutely certain this statement is FALSE	2	2	2	2	2	*I'm absolutely certain this statement is TRUE*
○	○	○	○	○	○	○

Click to write the question text

The risk described in this statement is SMALL	2	2	2	2	2	*The risk described in this statement is LARGE*
○	○	○	○	○	○	○

cb- first class

When flying on major airlines, you are more likely to be upgraded from economy to business class if you ask at the gate.

I'm absolutely certain this statement is FALSE	2	2	2	2	2	*I'm absolutely certain this statement is TRUE*
○	○	○	○	○	○	○

Click to write the question text

The benefit described in this statement is SMALL	2	2	2	2	2	*The benefit described in this statement is LARGE*
○	○	○	○	○	○	○

cb- lightning

In a thunderstorm, a hard-topped car can offer protection from lightning, as long as the occupants do not touch metal inside the car.

I'm absolutely certain this statement is FALSE	2	2	2	2	2	*I'm absolutely certain this statement is TRUE*
○	○	○	○	○	○	○

Click to write the question text

The benefit described in this statement is SMALL	2	2	2	2	2	*The benefit described in this statement is LARGE*
○	○	○	○	○	○	○

ch- intoxicated passenger

An intoxicated passenger could partially open the exit door on a commercial jetliner, causing the cabin to depressurize and the oxygen masks to deploy.

I'm absolutely certain this statement is FALSE	2	2	2	2	2	*I'm absolutely certain this statement is TRUE*
○	○	○	○	○	○	○

Click to write the question text

The risk described in this statement is SMALL	2	2	2	2	2	*The risk described in this statement is LARGE*
○	○	○	○	○	○	○

ch- lightning strikes

In the U.S., an average of 32 people are killed by lightning each year.

I'm absolutely certain this statement is FALSE	2	2	2	2	2	*I'm absolutely certain this statement is TRUE*
○	○	○	○	○	○	○

Click to write the question text

The risk described in this statement is SMALL	2	2	2	2	2	*The risk described in this statement is LARGE*
○	○	○	○	○	○	○

conspiracy intro

On the next two pages, you will find a series of statements asking about your general beliefs. You will be asked to indicate how likely you think that each statement is true, from certainly not true, to certainly true.

conspiracy items

I think that....

	certainly NOT true	extremely unlikely	very unlikely	unlikely	somewhat unlikely	undecided	somewhat likely	likely	very likely	extremely likely	certainly true
… there are secret organizations that greatly influence political decisions.	○	○	○	○	○	○	○	○	○	○	○
… politicians usually do not tell us the true motives for their decisions.	○	○	○	○	○	○	○	○	○	○	○
… events which superficially seem to lack a connection are often the result of secret activities.	○	○	○	○	○	○	○	○	○	○	○
	certainly NOT true	extremely unlikely	very unlikely	unlikely	somewhat unlikely	undecided	somewhat likely	likely	very likely	extremely likely	certainly true
… many very important things happen in the world, which the public is never informed about.	○	○	○	○	○	○	○	○	○	○	○
… government agencies closely monitor all citizens.	○	○	○	○	○	○	○	○	○	○	○

political success optimism items

I think that the political party I prefer will....

	certainly NOT true	extremely unlikely	very unlikely	unlikely	somewhat unlikely	undecided	somewhat likely	likely	very likely	extremely likely	certainly true
… control congress after the 2018 midterm elections.	○	○	○	○	○	○	○	○	○	○	○
...control the presidency after the 2020 elections.	○	○	○	○	○	○	○	○	○	○	○
	certainly NOT true	extremely unlikely	very unlikely	unlikely	somewhat unlikely	undecided	somewhat likely	likely	very likely	extremely likely	certainly true

	certainly NOT true	extremely unlikely	very unlikely	unlikely	somewhat unlikely	undecided	somewhat likely	likely	very likely	extremely likely	certainly true
...have significant legislative victories in the next three years.	○	○	○	○	○	○	○	○	○	○	○
...win major court cases in the next three years.	○	○	○	○	○	○	○	○	○	○	○

Demographics 1

Do you consider yourself an American?

How many letters are in the English language?

Your ethnicity:

Please enter your age in years

What is your height in feet and inches?

	Feet	Inches
fdf		

political items 1

Please indicate whether you agree or disagree, or are uncertain, with regard to each topic listed below:

	Agree	Disagree	Uncertain
socialism:	○	○	○
abortion rights:	○	○	○
waterboarding terror suspects:	○	○	○
prayer in public schools:	○	○	○
death penalty:	○	○	○
gay marriage:	○	○	○
illegal immigration:	○	○	○
theory of evolution:	○	○	○
small government:	○	○	○

	Agree	Disagree	Uncertain
globalization:	○	○	○
premarital sex:	○	○	○
pornography:	○	○	○
biblical truth:	○	○	○
pacifism:	○	○	○
patriotism:	○	○	○

political items 2

Please indicate whether you agree or disagree, or are uncertain, with regard to each topic listed below:

	Agree	Disagree	Uncertain
bomb cities controlled by terrorists:	○	○	○
drone attacks on terror suspects:	○	○	○
gun control:	○	○	○
obedience to authorities:	○	○	○
pollution control:	○	○	○
warrantless searches:	○	○	○
women's equality:	○	○	○
charter schools:	○	○	○
tax cuts:	○	○	○
military spending:	○	○	○
use nuclear weapons against threats to the US:	○	○	○
aid to foreign countries:	○	○	○
welfare spending:	○	○	○
compromise/negotiation with enemies:	○	○	○

politics items misc

Of the following major political parties in the U.S., which do you most strongly identify with?

- ○ Constitutional party
- ○ Libertarian Party
- ○ Green Party
- ○ Democratic party
- ○ Republican party

If you had to pick, which of these two candidates did you prefer in the 2016 general presidential election?

- Donald J. Trump
- Hillary Rodham Clinton

How strongly do you identify with the following political parties?

	Don't identify with at all	Strongly don't identify with	Somewhat don't identify with	Neutral	Somewhat identify with	Strongly identify with	Completely identify with
Republican Party	○	○	○	○	○	○	○
Libertarian Party	○	○	○	○	○	○	○
Green Party	○	○	○	○	○	○	○
Tea Party	○	○	○	○	○	○	○
Democratic party	○	○	○	○	○	○	○

moore questions

Who won the United States Senate special election in Alabama on Tuesday?

- **Doug Jones**
- **Barack Obama**
- **Bob Corker**
- **Herbert Price**
- **Roy Moore**
- **Not sure**

Did your preferred candidate win?

▾

How aware were you of of the Alabama Senate special election?

	Not aware at all	Mostly not aware	Somewhat not aware	Somewhat aware	Mostly aware	Completely Aware
.	○	○	○	○	○	○

How concerned were you about the outcome of the Alabama Senate special election?

	Not concerned at all	Mostly not concerned	Somewhat not concerned	Somewhat concerned	Mostly concerned	Completely concerned
.	○	○	○	○	○	○

In the time leading up to the Alabama Senate special election, how often did you read, listen, or watch about the race in the media?

demographics 2

Your gender?

- female
- male
- other / prefer not to say

When you look up on a clear day, what color is the sky?

- cereal bowl
- turkey
- blue
- train station
- orange juice
- laptop
- cardboard box

What is your approximate annual income in US Dollars (e.g. 27,000)?

Your highest level of education completed?

What sort of computer are you using to participate?

In all honesty, did you pay close attention during this study? (Please answer honestly--your answer will improve the quality of the study, but will not affect your payment or Mechanical Turk rating in any way.)

turker code

Your survey complete code is HG87F2

Important!
You need the code above to claim your MTurk payment

Your survey complete code is Z5NH2Y

Important!
You need the code above to claim your MTurk payment

Your survey complete code is 8KMIYI

Important!
You need the code above to claim your MTurk payment

Your survey complete code is 5CMR5D

Important!
You need the code above to claim your MTurk payment

Your survey complete code is 3YQA7V

Important!
You need the code above to claim your MTurk payment

debrief

Thank you for participating!

This study is intended to investigate how social attitudes and abstract reasoning affect the evaluation of information. Please keep in mind that, although some of the statements that you evaluated are true, many of them are false.

If you would like to be informed via email as to which of the statements are true and which are false, please enter your email address in the box below, and we will email you after this project has been completed; your email address will be stored separately from your responses to all questions on this survey. After the requested information is sent, all email addresses will be deleted.

(TOTALLY OPTIONAL) Your email address:

Thank you again, very much, for your assistance. If you have any questions or concerns about this research, please feel free to contact me.

Daniel M.T. Fessler, Ph.D, Professor

Dept. of Anthropology, UCLA

Los Angeles, CA 90095-1553

Email: dfessler@anthro.ucla.edu

Tel. (310) 794-9252

You can also contact the OHRPP (formerly OPRS), Office of Human Research Protection Program, UCLA, 11000 Kinross Avenue, Suite 102, Box 951694, Los Angeles, CA 90095-1694, (310) 825-7122.

Powered by Qualtrics
